# KDM6A mutations promote acute cytoplasmic DNA release, DNA damage response and mitosis defects

**DOI:** 10.1186/s12860-021-00394-2

**Published:** 2021-10-26

**Authors:** J. Koch, A. Lang, P. Whongsiri, W. A. Schulz, M. J. Hoffmann, A. Greife

**Affiliations:** 1 Department of Molecular Physical Chemistry, Heinrich-Heine-University Duesseldorf, Duesseldorf, Germany; 2 Department of Cardiology, Pulmonology, and Vascular Medicine, Medical Faculty, Heinrich-Heine-University Duesseldorf, Duesseldorf, Germany; 3 Department of Urology, Medical Faculty, Heinrich-Heine-University Duesseldorf, Duesseldorf, Germany; 4 Department of Biochemistry and Microbiology, Faculty of Pharmaceutical Sciences, Chulalongkorn University Bangkog, Bangkok, Thailand

**Keywords:** KDM6A mutations, Mitosis defects, DNA damage, Nuclear integrity

## Abstract

**Background:**

KDM6A, encoding a histone demethylase, is one of the top ten mutated epigenetic cancer genes. The effect of mutations on its structure and function are however poorly characterized.

**Methods:**

Database search identified nonsense and missense mutations in the N-terminal TPR motifs and the C-terminal, catalytic JmjC domain, but also in the intrinsically disordered region connecting both these two well-structured domains. KDM6A variants with cancer-derived mutations were generated using site directed mutagenesis and fused to eGFP serving as an all-in-one affinity and fluorescence tag to study demethylase activity by an ELISA-based assay in vitro, apoptosis by FACS, complex assembly by Co-immunoprecipitation and localization by microscopy in urothelial cells and apoptosis by FACS.

**Results:**

Independent of the mutation and demethylase activity, all KDM6A variants were detectable in the nucleus. Truncated KDM6A variants displayed changes in complex assemblies affecting (1) known interactions with the COMPASS complex component RBBP5 and (2) KDM6A-DNA associated assemblies with the nuclear protein Nucleophosmin. Some KDM6A variants induced a severe cellular phenotype characterized by multiple acute effects on nuclear integrity, namely, release of nuclear DNA into the cytoplasm, increased level of DNA damage indicators RAD51 and p-γH2A.X, and mitosis defects. These damaging effects were correlated with increased cell death.

**Conclusion:**

These observations reveal novel effects of pathogenic variants pointing at new specific functions of KDM6A variants. The underlying mechanisms and affected pathways have to be investigated in future research to understand how tumor cells cope with and benefit from KDM6A truncations.

**Supplementary Information:**

The online version contains supplementary material available at 10.1186/s12860-021-00394-2.

## Background

In the past decade, deep sequencing approaches have revealed mutations in many genes encoding epigenetic modifiers, prominently *KDM6A* [1]. *KDM6A* is frequently mutated in several cancer types, especially in urothelial cancer [2–4], and also in the hereditary Kabuki syndrome [5]. The human H3K27-demethylase KDM6A, also referred to as *Ubiquitously transcribed Tetratricopeptide repeat, X chromosome* (UTX), specifically removes the ε-di/trimethylation on lysine 27 of histone H3, a repressive histone modification [6]. KDM6A interacts with other chromatin-modifying protein complexes. In particular, interactions with KMT2C/D-complexes (COMPASS) with the core components WDR5, RBBP5, ASH2L and DPY30 [7] appear to be demethylase-dependent at bivalent H3K27me3/H3K4 domains [8]. KDM6A binding at promoter sequences of actively transcribed genes [9] is mainly demethylase-independent and involves interactions with the SWI/SNF chromatin remodeling complex [10], coactivators CBP/p300 [11], but also components of the transcription elongation machinery, like the SUPT6H-RNA Polymerase II complex [12].

The KDM6A protein has three functionally and structurally relevant regions conserved among several vertebrates. These include the eight N-terminal tetratricopeptide repeats (TPRs, aa 92–385 in human), which represent a common protein interaction motif [13]. Interestingly, the human KDM6A TPR3 and TPR5 conform poorly to the consensus (1–4%) as highlighted in Figure S1, but secondary structure predictions indicate that the essential helix-turn-helix motif is still preserved. The well-characterized C-terminal and highly conserved, catalytically active Jumonji C domain (JmjC) is important for histone H3K27me2/3-recognition, binding and demethylation [14]. In addition to the JmjC core (approx. aa 1095–1260), flanking regions termed pre- and post-JmjC are essential for its catalytic activity (approx. aa 880–1094, 1261–1395). The two well-structured domains, TPR and JmjC, are connected by an intrinsically disordered region (IDR) [15]. Interestingly, a high number of other chromatin-associated proteins, such as Nucleophosmin, p300 and KMT2A-D, possess such flexible sequences, which seem to be common and crucial motifs for interactions with proteins and DNA/RNA [16].

In cancer, *KDM6A* mutations can be found throughout the whole coding region, most prevalently within the functionally relevant domains. Knowing the precise effect of mutations on the functionality of KDM6A is key to fully understand the pathogenic effects resulting from impairments of this protein and to develop strategies for targeted therapies. Therefore, we developed a workflow to systematically and simultaneously follow changes of the intrinsic properties of KDM6A substitution and truncation variants, using eGFP as an all-in-one fluorescence marker and affinity tag. We characterized the impact of KDM6A mutations on (i) demethylase activity by an ELISA-based demethylase assay, (ii) protein stability by western blotting, (iii) intracellular (co-)localization by immunofluorescence, scatterplot analysis and cellular back-mapping, (iv) complex assemblies by co-immunoprecipitation (Co-IP) and (v) cell death using an Annexin V based flow cytometry analysis.

## Results

### Generation of amino acid substitution and truncated variants of KDM6A based on the mutational landscape of KDM6A in tumor tissues and cell lines

**Fig. 1:**
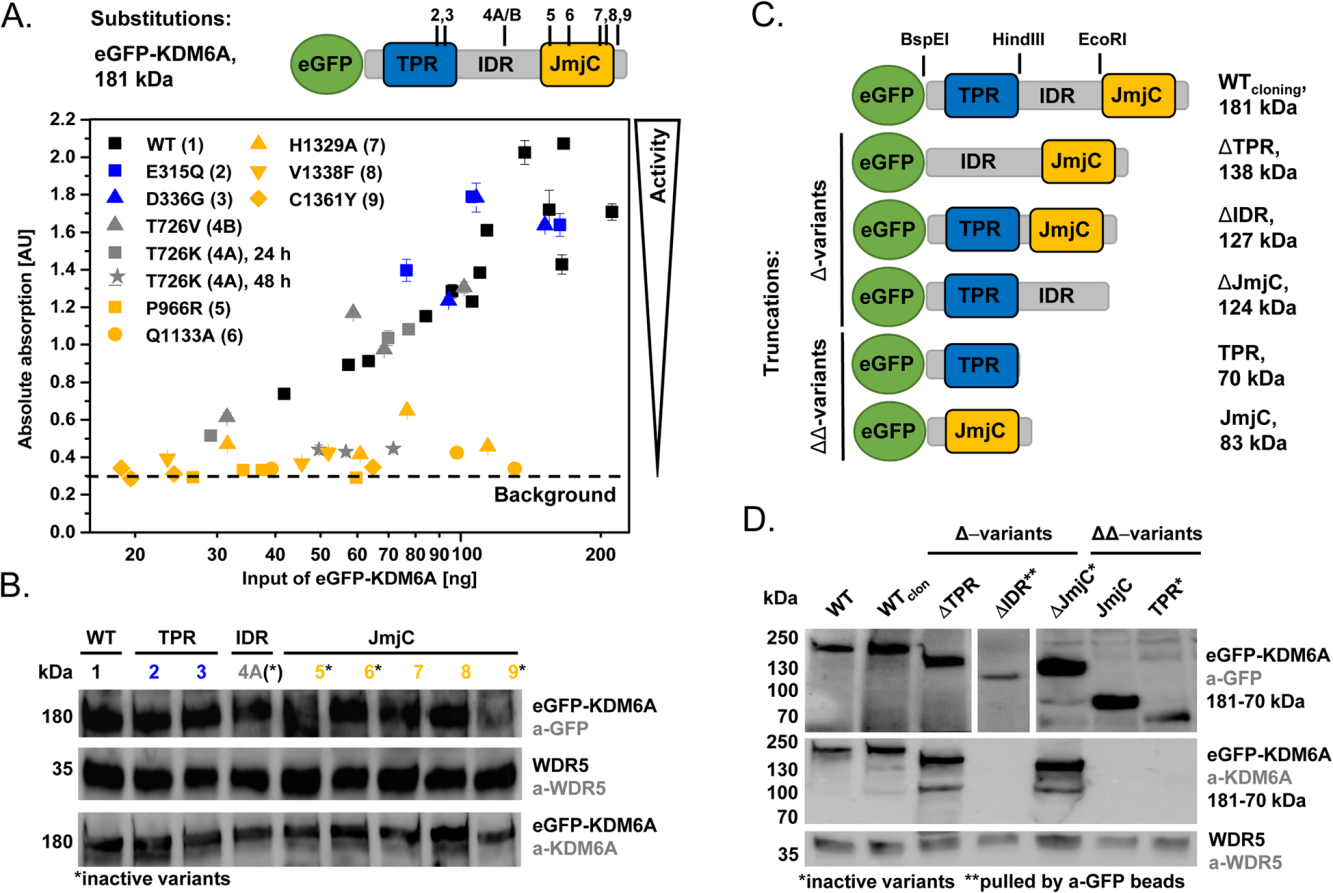
Protein expression and activity of KDM6A substitution and deletion variants. **A** Results of the ELISA-based activity assay of KDM6A WT and substitution variants are displayed as normalized absorption on the y-axis, which positively correlates with demethylated product. Calculated amount of protein is depicted on the x-axis in logarithmic scale. WT (black) and substitutions variants in TPR (blue) and IDR (grey) generate more demethylated product with rising input amounts. Substitutions affecting the JmjC domain (yellow) remain mostly at baseline levels. T726K activity (#4A) increases with input amount at 24 h (grey boxes) but not at 48 h (grey stars). Error bars represent the standard deviation between the triplicates of each individual experiment (*n* = 4–18, depending on variant) **C** Graphic display of KDM6A truncation variants. **B/D** Western blot showing all substitution and deletion variants 48 h after transient transfection, detected with a-eGFP (CST) and a-KDM6A (CST) antibodies, as well as WDR5 (CST) as lysate control. Variants with IDR deletions are not detectable with the a-KDM6A antibody, as it recognizes epitopes within the IDR. The ΔIDR variant was enriched via IP. Color code blue-grey-yellow according to the site of mutation (as depicted in **A** and **C**)

Based on 64,668 tested samples from 44 tissue types, 2496 unique mutations are listed for *KDM6A* in COSMIC v92 (GRCh 38, November 2020). Among the cancer tissues, meninges and the urinary tract exhibit the highest mutation frequency in each more than 30% of the tested cancer samples (411/1336 cases for UC). The three most frequent point mutations across all tissues are the synonymous mutation Q1037 = (c.3111G > A) in the JmjC domain, the missense mutation T726K (c.2177C > A) and the truncating Q555* (c.1663C > T), both in the intrinsically disordered region. We selected T726K as a hotspot substitution variant as well as substitution mutations typically found in urothelial cancer cell lines and tissues: E315Q (located in TPR6) and D336G (TPR7), P966R (loop in a beta sheet cavity containing the active center), V1338F (JmjC, zinc-binding) and C1361Y (JmjC, zinc-binding). To compare the impact of these substitution mutations, we additionally selected the variants Q1133A (H3-tail interaction, JmjC) and H1329A (hydrophobic patch near active center, JmjC) known to be catalytically inactive [14]. Positions P966, Q1133, V1338 and C1361 are conserved in the KDM6A zebrafish ortholog and the closest functional KDM6A paralogs KDM6B and KDM6C. We created all substitution variants by site-directed mutagenesis using eGFP-KDM6A as a template (Fig. 1A). As nonsense mutations make up nearly one quarter of all point mutations and generate truncated variants with partial losses of the IDR and JmjC, we established a set of truncated variants, each with an eGFP-tag fused to the N-terminus: ΔTPR, ΔIDR, ΔJmjC, TPR (= ΔIDR/ΔJmjC) and JmjC (= ΔTPR/ΔIDR) (Fig. 1C). These variants were transiently transfected into urothelial cancer cell lines with wildtype (SW-1710) or mutated (T-24) KDM6A/KMT2C/D proteins to study the influence of the endogenous proteins on the cellular response after reintroduction. All KDM6A substitution and deletion variant proteins were detectable at the expected sizes (Fig. 1). In a second step, we analysed all variants for their demethylation activity. KDM6A JmjC and the flanking zinc-binding domain are known to recognize and bind several amino acids between H3R17-H3T32 of the H3K27 di- and tri-methylated N-terminal histone tail to ensure substrate specificity [14]. Consequently, mutations in the JmjC and flanking domains have a high potential to impede or even abolish the catalytic activity. However, it is unknown to what extent mutations in the TPR and IDR might contribute to KDM6A demethylase activity.

### KDM6A demethylase activity is strongly affected by substitutions and deletions within the JmjC domain

To assess the demethylase activity of KDM6A variants, we established an ELISA-based H3K27me3 demethylation assay to screen for the catalytic activity of all truncated and substitution variants. The assay was carried out with the purified protein attached to GFP-dynabeads. We used fluorescence emission of the eGFP to determine and normalize the amount of protein before and after binding to dynabeads (Figure S2A-C). The activity of the variants was measured by colorimetric readout of the demethylated products and subsequent fitting via 4PL-regression. The eGFP-KDM6A WT species served as a reference point for demethylase activity (Figure S2D-G). The activity of substitution variants was assessed in a quantitative manner, yielding the specific activities summarized in Table 1. As shown in Fig. 1A, eGFP-KDM6A variant T726V featured a demethylase activity in the range of WT. Note that TPR substitutions E315Q and D336G had a slightly higher activity. Interestingly, activity in T726K decreased time-dependently within 2 days after transfection (Figure S2I). H1329A and V1338F had strongly reduced activity, while P966R, Q1133A and C1361Y were considered as non-active.

**Table 1:** Specific activity of substitution variants

No.	KDM6A variant, affected domain	Specific activity [10^**− 3**^ μmol min^**− 1**^ mg^**− 1**^]
1	WT	2.15 ± 0.24
2	E315Q, TPR	3.96 ± 0.58
3	D336G, TPR	3.76 ± 0.89
4	T726K, IDR	0.69 ± 0.10
5	T726V, IDR	1.54 ± 0.11
6	P966R, JmjC	< 0.07 ± 0.03
7	Q1133A, JmjC	< 0.09 ± 0.02
8	H1329A, JmjC	0.28 ± 0.05
9	V1338F, JmjC	0.29 ± 0.03
10	C1361Y, JmjC	< 0.12 ± 0.03

Specific activity of KDM6A variants depends strongly on the mutation site. Mutations affecting the JmjC domain impair catalytic activity, whereas TPR substitution mutation enhanced activity. The IDR mutation T726K showed time-dependent diminution of activity. Specific activity was calculated as described in Figure S2

The demethylase activity of the truncated KDM6A variants was assessed in a qualitative manner since changes in the fluorescence emission spectra of the truncated variants did not allow proper quantification of the protein amount used in the assay (Figure S2C). As expected, experiments with truncated (Δ- and ΔΔ-) variants yielded activity only when the JmjC domain was preserved (Figure S2H).

### KDM6A single substitutions do not alter nucleoplasmic localization

**Fig. 2:**
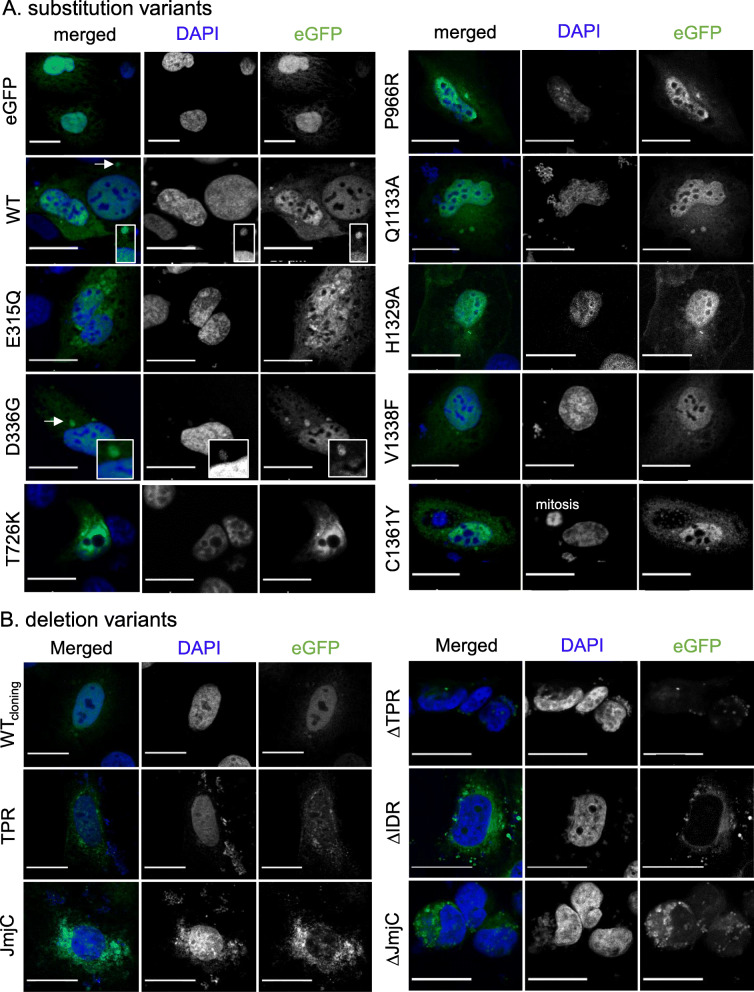
Localization of substitution and deletion variants. **A** Localization of transiently transfected KDM6A substitution variants in SW-1710 (for T-24 see Figure S4) using confocal microscopy and post-processing with HuygensPro 20.08. All variants localized to the nucleoplasm and cytoplasm. Occasionally, KDM6A positive micronuclei and cytoplasmic DNA were observed for all variants including wildtype (arrows). **B** Abnormal localization pattern compared to the eGFP-KDM6A WT, WT_cloning_ and substitution variants: Leakage of DNA from the nucleus and subsequent extranuclear DNA patches are common features with all truncated variants. Scale bar is 20 µm

In SW-1710 and T-24 cells eGFP-KDM6A WT was located in the nucleoplasm and in the cytoplasm, sometimes with a tendency to cytoplasmic speckle formation (dependent on the dose of the transient overexpression). All substitution variants showed cytoplasmic and predominantly nucleoplasmic localization with different degrees of cytoplasmic speckle formation, as well as weak accumulation around the nucleoli (Fig. 2A and Figure S4A). To rule out unspecific or GFP driven localization of transfected KDM6A, we analyzed free eGFP controls in SW-1710, T-24 and HBLAK cells (Fig. 2A, Figures S3/4). Free eGFP as well as eGFP-KDM6A shows both cytoplasmic and nuclear localization. The main difference between eGFP and eGFP-KDM6A is the additional presence of free eGFP in nucleoli while eGFP-KDM6A is exclusively found nucleoplasmic. The localization of eGFP-KDM6A is similar to that of the endogenous KDM6A in these cell lines by immunostaining using two different KDM6A antibodies (sc-514,859 and CST-3351, Figure S3).

**Fig. 3:**
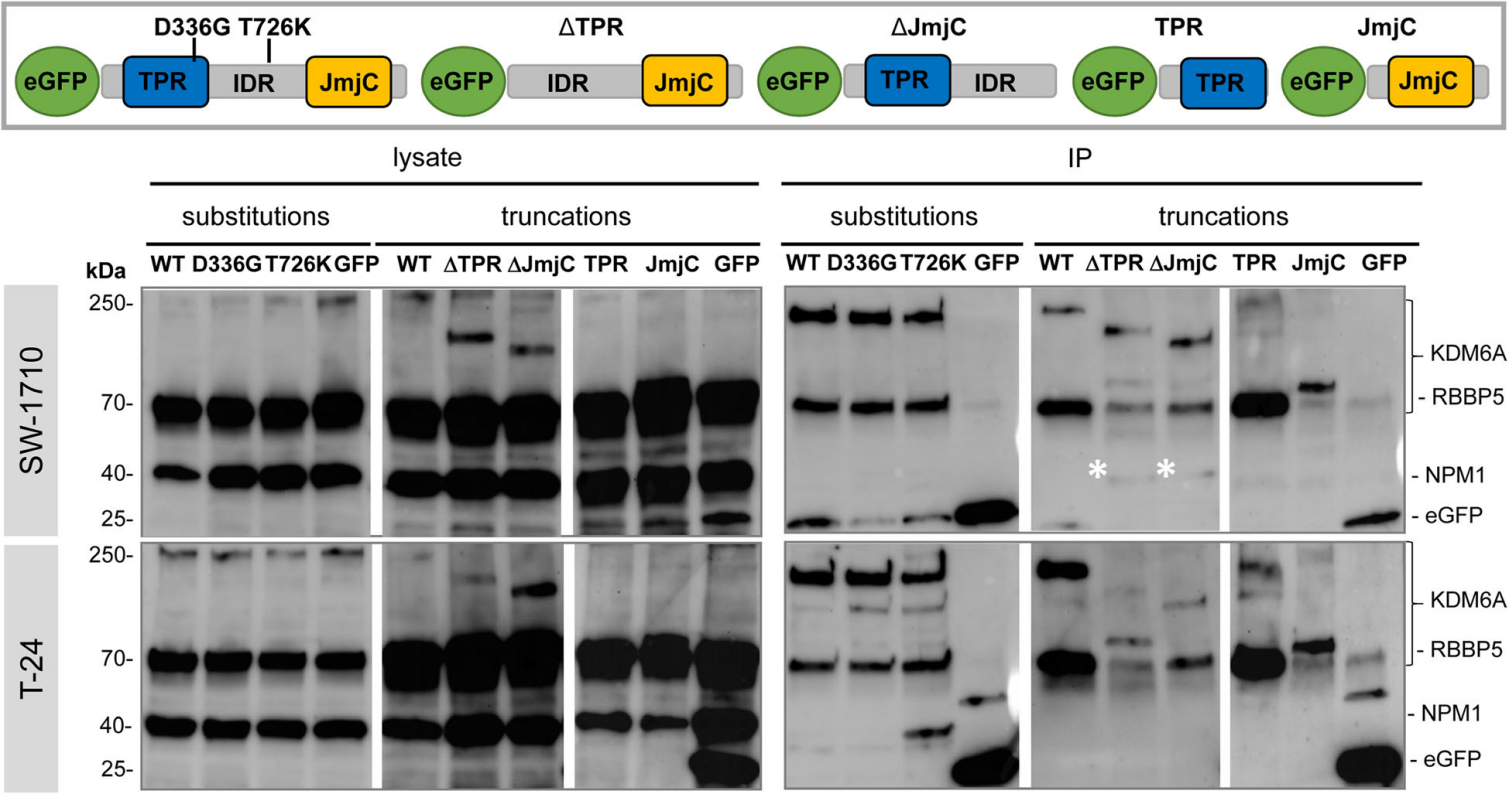
RBBP5 and NPM1 interaction analysis. Top section: KDM6A variants used in the interaction study. Mid/Bottom section: IP of KDM6A variants with RBBP5 and NMP1 in SW-1710 and T-24 (*n* = 2–7). The cleared cell lysates (left section) indicates GFP, RBBP5 and NMP1 protein levels after transfection of KDM6A WT, variants and the eGFP control. For SW-1710 (right section, mid) and T-24 (bottom), IP was used to pull RBBP5 with eGFP-KDM6A WT, D336G, T726K, ΔTPR, ΔJmjC, TPR, JmjC and the control eGFP. In SW-1710, NPM1 is slightly enriched in the IP in the eGFP-KDM6A ΔTPR and ΔJmjC variants (white stars), whereas in T-24, NPM1 is enriched in eGFP-KDM6A T726K variant (*n* = 1). The TPR and RBBP5 signals are separately shown in Figure S5, as well as controls and raw Western Blot images

KDM6A transport into the nucleus depends largely on the KMT2C/D COMPASS complex [17]. Therefore, the observation that KDM6A WT and all substitution variants localized in the nucleus suggests functional nuclear import, as well as interaction with the COMPASS complex. Notably, a recent study [17] found that KDM6A TPR mutations, among them the D336G variant, predominantly localized in the cytoplasm in stably transfected HeLa cells. We performed a pull-down experiment of KDM6A WT, D336G and T726K variants with RBBP5 to test for impaired association with the KMT2C/D complex. RBBP5 is a core component of KMT2 complexes that directly interacts with ASH2L, WDR5 and DPY30 within the WRAD complex to bind specific chromatin sequences, stabilize and activate the methyltransferase activity of KMT2 proteins. RBBP5 was pulled down with all three KDM6A variants to similar extents, but not with the eGFP control in both urothelial cancer cell lines (Fig. 3), suggesting that they are present in the same complex. Furthermore, perinuclear KDM6A speckles also stained DAPI-positive, indicated newly formed micronuclei. Micronucleus formation, an indicator of genomic instability, is however common in urothelial cancer cell lines. An increased tendency for DNA release was identified with some KDM6A substitution variants, which colocalized at cytoplasmic DNA sequences (Fig. 2, catalytically impaired variants). This phenomenon was much stronger in KDM6A truncation variants.

#### KDM6A truncated variants cause severe nuclear damage

Compared to the eGFP and eGFP-KDM6A WT controls, deletion of any functional domain resulted in a heterogenous cellular response with respect to subcellular distribution, localization and nuclear integrity. KDM6A variants displayed a weak cytoplasmic and nucleoplasmic localization and in some varinats presented a speckled, perinuclear distribution of the transfected protein associated with nuclear defects shown in Figs. 2 and S4. Cellular responses in both cell lines were comparable. For comparision, we transfected the KDM6A TPR and JmjC variants into HBLAK cells, a non-transformed urothelial cell line, and observed similar damaging effects. Nuclear DNA, which was excessively released into the cytoplasm, colocalized with KDM6A truncation variants. These results indicate the intrinsic ability of all variants to bind chromatin, either directly through the JmjC or indirectly via other protein-protein interactions by TPR-containing variants. The presence or absence of the intrinsically disordered region (IDR) strongly affects stability and solubility of KDM6A. All variants principally localized to the nucleus, but (to various extents) showed anomalies like partial nuclear redistribution to nucleoli or accumulation in perinuclear DNA-associated speckles. These observations raised two further questions, namely (1) how deletions of one or two KDM6A domains impair functional interactions with known interacting proteins such as RBBP5, the KMT2C/D (COMPASS) complex and (2) whether Nucleophosmin (NPM1), a prominent shuttling and chaperone protein found especially in nucleoli, may be involved in KDM6A interactions with these organelles. To address these questions, we performed protein immunoprecipitation (Co-IP) and co-staining of transiently transfected KDM6A variants with NPM1 and RBBP5.

### Full-length KDM6A is needed for maximal binding of the COMPASS-complex core component RBBP5

First, we tested for RBBP5 interaction with KDM6A by Co-IP experiments 48 h post transfection of selected KDM6A truncation variants in T-24 and SW-1710 (Fig. 3). We had previously shown that RBBP5 was enriched in KDM6A-tagGFP2 Co-IPs in different urothelial cancer cell lines [18]. Interestingly, we observed that for maximum interaction with RBBP5 all KDM6A domains are needed (Fig. 3). Deletion of any domain impaired RBBP5 binding. Specifically, TPR-containing variants (ΔJmjC and TPR, Figure S5) and to some extent IDR-containing variants (ΔTPR) bound RBBP5 to some degree, but the JmjC domain alone did not at all.

Intriguingly, proteomics analysis from two KDM6A-tagGFP2 stably transduced urothelial cancer cell lines showed a significant enrichment of nucleolar proteins, such as Nucleolin, NPM1 and ribosomal subunits, in addition to histone variants (Figure S6). This enrichment was not seen with eGFP alone. However, in Co-IP experiments NPM1 could only be significantly enriched with the KDM6A T726K mutant in T-24 (Fig. 3), whereas other selected KDM6A variants failed to interact or gave very weak bands (ΔTPR and ΔJmjC, Fig. 3 white stars). Therefore, we searched for a complementary approach to detect associations of KDM6A with NPM1.

Scatterplots are widely used to detect colocalization. Typically, the intensities of two channels are plotted in a xy-diagram and the resulting populations elucidate on colocalization, which is displayed in Pearson (P between − 1 and 1) or channel-wise Manders values (M1, M2 between 0 and 1). However, one drawback of such values is that the connection between scatterplot populations and the spatial cellular information is lost. For this reason, we used images from a single z-slice and generated scatterplots to identify populations of interest, mapped them back to the respective cellular compartment using the freely available Fiji plugin ScatterJ [19] and compared the outcome with line profiles through the corresponding cellular compartments. By this approach, we identified localization changes among the KDM6A variants in SW-1710 cells.

### KDM6A WT and NPM1 form nucleoplasmic populations

**Fig. 4:**
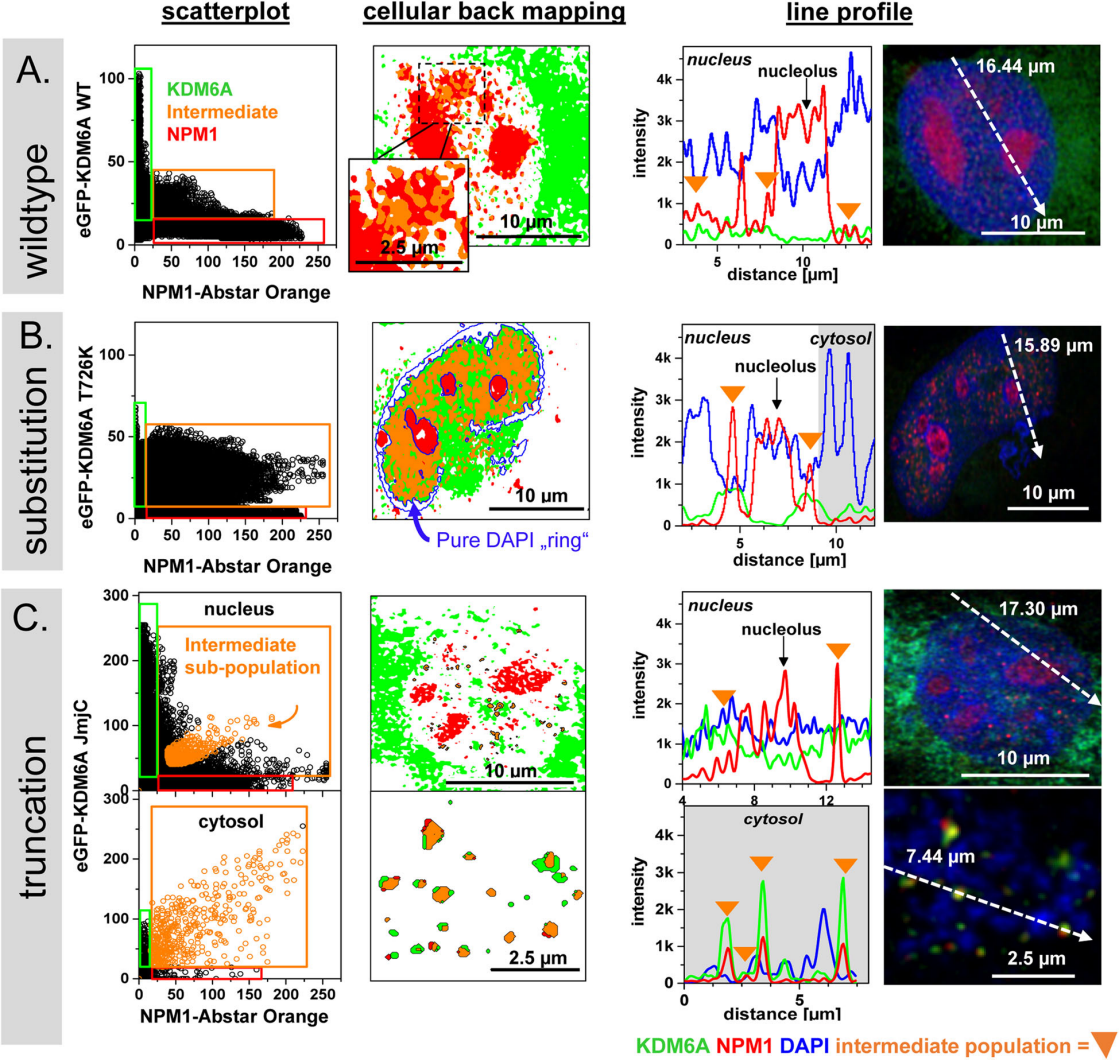
Cellular back mapping identifies co-localizing NPM1-KDM6A protein populations in nucleoplasm and at extranuclear DNA. **A-C** Left panel: Scatterplot presentation of KDM6A (green box) and NPM1 (red box) and one intermediate population representing a KDM6A-NPM1 assemblies (orange box). Middle panel: All three populations were mapped back to the cellular compartment. Right panel: Line profile through the nucleus as indicated by an arrow in the image. DAPI (blue), NPM1 (red) and KDM6A variants (green) signals are overlaid. The orange triangles indicate the intermediate populations from the scatterplot. **A** Analysis of KDM6A WT and NPM1. **B** Analysis of KDM6A T726K and NPM1. **C** Analysis of KDM6A JmjC variant with NPM1 in the nucleus and at extranuclear DNA. Scatterplot and back mapping analysis for KDM6A variants and DAPI is shown in Figure S7

As shown in the scatterplot for KDM6A WT and NPM1 (Fig. 4A, left panel), two unique fractions appeared at the y- or x-axis, representing signals in only one of the two channels. The corresponding eGFP-KDM6A WT-only fraction is shown in green and the NPM1-only fraction in red. A third fraction with green and red signals of different intensities is shown in orange and named from here on “intermediate” fraction from here on. Cellular back mapping of the selected populations (Fig. 4A, middle panel), clearly showed the KDM6A WT-only signal predominantly in the cytoplasm and only a minor fraction in the nucleoplasm. As expected, the NPM1-only signal was present in nucleoli and in nucleoplasm. The intermediate fraction was always associated with the NPM1-only fraction at the nucleoli rims and within the nucleoplasm, indicating a dynamic exchange of NPM1-only, mixed complexes and KDM6A WT-only fractions at specific sites within the nucleus. The line profile through the nucleus with DAPI as a DNA indicator (Fig. 4A, right panel) corroborates these findings: High NPM1 (red) signal intensities were exclusively found in nucleoli, whereas green-red overlapping signals might represent KDM6A WT-NPM1 assemblies.

### The T726K hotspot mutant is absent from nucleolamina and additionally forms cytoplasmic complexes

By comparing the scatterplot presentations from T726K or WT KDM6A and NPM1, we observed a much broader intermediate fraction with similar KDM6A-T726K signal intensities but higher NPM1 intensities (Fig. 4B), which was also visible in the line profile. By cellular back mapping, we identified the intermediate population in the nucleoplasm and in the cytoplasm, where KDM6A T726K (green population) was also detectable. In contrast, the nucleoplasmic NPM1-only fraction was completely absent with this mutant. In the original image (Figure S7A), DAPI staining clearly indicated DNA release into the cytoplasm. Scatterplot analysis of KDM6A T726K with DAPI (Figure S7B) showed KDM6A T726K associated to DNA in the nucleus and at the released DNA (population 3). Furthermore, we identified a DAPI-only fraction (population 4), which was also negative for NPM1 at the nuclear lamina.

### Truncated KDM6A variants likewise form complexes with NPM1 at extranuclear DNA segments

As described above, truncated KDM6A variants elicited more severe nuclear DNA release and nuclear damage (Fig. 2). As an example, we selected KDM6A JmjC and KDM6A TPR variants (Fig. 4C) for further analysis. Scatterplot analysis and line profiling of the truncated variants with NPM1 clearly indicated an enriched, colocalizing intermediate fraction at extranuclear DNA segments. As observed before, KDM6A JmjC and TPR variants were also localized to the cytoplasm. In contrast to KDM6A WT, these variants were always associated with DNA, as shown by DAPI-KDM6A scatterplot analysis and cellular back mapping approaches (Figure S7B/C). By combining these approaches, we identified KDM6A-NPM1 assemblies, which were hardly detectable by Co-IP/WB analysis (Fig. 3), which could be caused by experimental characteristics (proportion of extranuclear DNA segments is too little compared to whole cell lysate) and resolution limits of the respective experiment (here fluorescene imaging is superior to Co-IP). Importantly, scatterplot analysis and the cellular back mapping approach highlighted differences among the wildtype, substitution and truncated KDM6A variants. The T726K variant represents a non-wildtypic phenotype with reduced enzymatic activity in a time-dependent manner and sometimes even DNA release. The severe phenotype of the truncated variants is characterized by a massive DNA release and forms nearly equal complexes with NPM1.

#### The severe phenotype of KDM6A truncation variants is characterized by mitotic defects, DNA release and significantly decreased cell viability

**Fig. 5:**
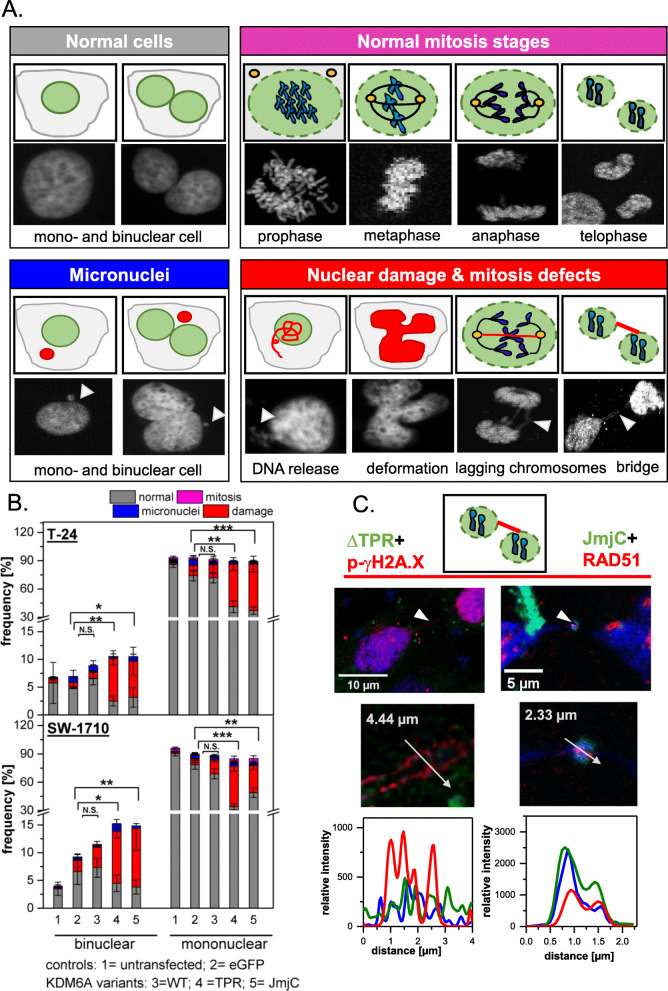
Truncated KDM6A variants promote a significant decrease in cells with normal phenotypes in T-24 and SW-1710 cells. **A** Scoring criteria used to quantifiy the occurrence of nuclear damage. **B** Frequency of different phenotypes scored as normal interphase (grey) and mitosis (pink), micronuclei (blue) and damage (red) in bi- or mononuclear T-24 and SW-1710 untransfected (UT, 1) cells or transfected with control eGFP (2), eGFP-KDM6A variants WT (3), TPR (4) and JmjC (5) (for simplicity abbreviated as WT, TPR and JmjC). In mononuclear and binuclear cells, the proportion of normal cells decreased significantly for both the eGFP-KDM6A TPR and JmjC compared to eGFP and eGFP-KDM6A WT. Cells with micronuclei were not significantly enriched. This was largely due to an increase in damaged cells. Overall, we observed a non-signifcant trend towards an increased number of binucleated cells. T-test using two-tailed hypothesis, significance levels: * = *P* ≤ .05, ** = *P* ≤ 0.01, *** = *P* ≤ 0.001, see Table S3 for *P*-values. **C** Line profiles through lagging chromosomes and the “knot-like structures” of the chromatin bridges found in samples transfected with eGFP-KDM6A TPR (left) or JmjC (right) indicating overlapping signal intensities of KDM6A variants (green) with DNA damage markers RAD51 and p-γH2AX (red)

**Fig. 6:**
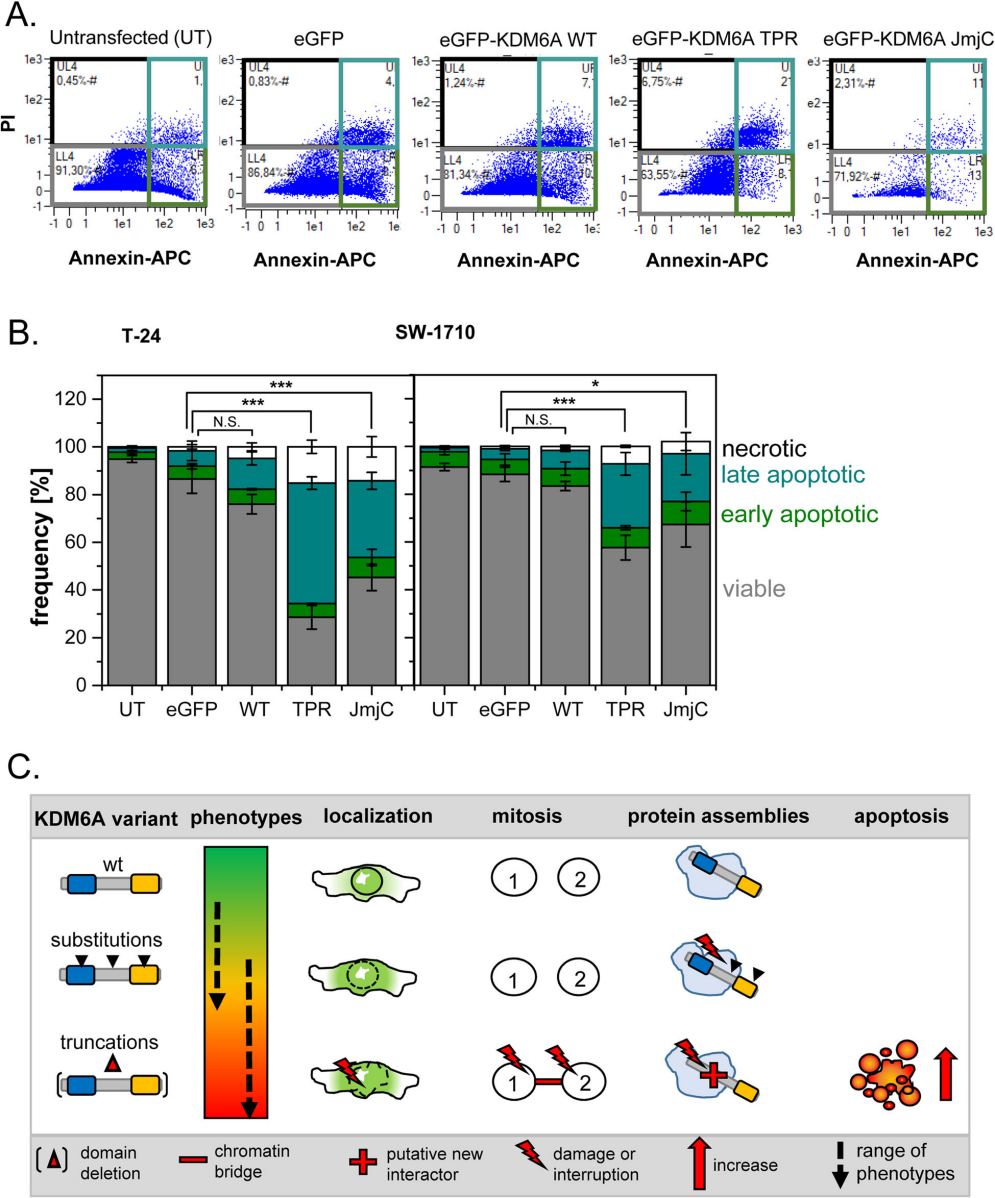
Truncated KDM6A variants decrease the amount of viable in T-24 and SW-1710 cells and exhibit mitosis errors. **A** FACS analysis in Annexin V based apoptosis assay. Transfected eGFP positive cells were gated based on the threshold obtained from untransfected cells (UT), which were then used to plot PI (for membrane permeability) against Annexin-V-APC (apoptosis marker). The plot was divided into four quadrants, representing the viable population (lower left, grey), early apoptosis (lower right, green), late apoptosis (upper right, dark cyan) and necrosis (upper left, black). **B** Statistics derived from triplicate measurements. eGFP-KDM6A TPR and JmjC show a significant decrease in cell viability in comparision to the eGFP control in both cell lines. T-test using two-tailed hypothesis, significance levels: * = *P* ≤ .05, ** = *P* ≤ 0.01, *** = *P* ≤ 0.001, see Table S3 for detailed *P*-values. **C** Graphic summary of cellular phenotypes observed with KDM6A mutation variants, depicting the impact on localization, mitosis, apoptosis and protein assemblies

As DNA release has been observed, we analysed whether the typical indicators for a DNA damage response are activated after introduction of KDM6A truncation variants, namely accumulation of phospho-γH2A.X and RAD51. Especially JmjC containing variants colocalize with both markers at chromatin bridges (Fig. 5C). Furthermore, we detected elevated levels of phospho-γH2A.X with truncated KDM6A variants, whereas overall phospho-γH2A.X levels were unchanged in KDM6A WT transfected cells, even in cells with strongly overexpressed KDM6A WT protein (Figure S8). Visual inspection of phospho-γH2A.X in cells with truncated variants revealed defects in mitosis (Fig. 5). These defects occurred in anaphase as lagging chromosomes, multiple fragmentation events, in telophase and cytokinesis by persisting chromosome bridges and accumulation of DNA damage sites at chromosome bridges. To quantify our observations, we established a scoring system to determine the amount of cells with severe phenotypes per variant (Fig. 5A). Here, we discriminated between mono- and binucleated cells and quantified cells with damages (cytoplasmic DNA release, extreme nuclear deformation, lagging chromosomes and chromosome bridges), micronuclei, normal interphase or mitosis. Both KDM6A deletion variants, TPR and JmjC, elicited a significant increase in DNA damage in mono- and binucleated cells (Fig. 5B). We observed a trend for cells transfected with eGFP-KDM6A WT, TPR and JmjC to be detected in binuclear cells. To evaluate whether the nuclear damage promotes apoptosis, we performed an Annexin V-based apoptosis assay 48 h post transfection. The FACS measurements indicate that cell viability significantly decreases in eGFP-KDM6A TPR and JmjC variants in both urothelial cancer lines (Fig. 6A/B). This mirrors the results of the scoring experiments very well, pointing towards a direct connection between the nuclear damage and apoptosis induction.

## Discussion

In this study, we developed and utilized a comprehensive analysis tool kit to understand the relationship and interplay of known and predicted regulatory features of the multi-domain protein KDM6A. We demonstrate how single substitution mutations and deletions of the main three functional and regulatory domains, TPR, IDR and JmjC, affect the intrinsic properties of the target protein and its interactions with the cellular environment. We could show that mutations within the JmjC domain affected the catalytic activity. Although nuclear localization changed little, interactions with RBBP5 and NPM1 were affected, especially in truncated variants, where we observed a cellular mechanism to dispose of harmful KDM6A variants, namely by cytoplasmically released DNA-KDM6A complexes. Harmful KDM6A variants cause mitosis defects and DNA damage which promotes cell death.

First, we developed an ELISA-based demethylase assay suited for use with eGFP-tagged KDM6A variants. Our experiments revealed, that substitution variants within the JmjC (catalytic domain) possessed reduced or abolished demethylase activity, especially if amino acids involved in peptide recognition, peptide binding or stabilizing were changed. Remarkably, the demethylase activity was also reduced in the IDR-variant T726K, but not in T726V. Therefore, we predict a unique functionality for the amino acid position K726 (Table 1, Figure S2). Among the selected cancer-associated point mutations, the hotspot mutation T726K is the third most frequently listed one in Cosmic v92 across all tissues and was the only substitution variant yielding a time-dependently lowered demethylase activity. We predicted and tested K726 as a possible methylation site, but mass spectrometry analysis did not show any PTM at K726. The demethylase activity of truncated KDM6A variants was determined by the presence or absence of the JmjC domain. As expected, KDM6A JmjC, ΔTPR and ΔIDR exhibited demethylase activity, whereas TPR and ΔJmjC did not (Figure S2). However, removal of other domains negatively affected protein stability and solubility, especially for KDM6A ΔIDR (Table S1). While those variants with N-terminal or central truncations are mostly artificial, C-terminally truncated variants generated by nonsense mutations make up almost a quarter of all listed *KDM6A* mutations in COSMIC v92. A prevalent nonsense mutation is Q555*. Its prevalence may be partly explained by the observation that it represents a hotspot for APOBEC3-mediated mutations [20], which are frequent in multiple cancer types including bladder cancer [21]. Functionally, the Q555* variant fully lacks the JmjC domain and has a partial deletion of the IDR. Such IDR/JmjC nonsense mutations would abolish demethylase activity by truncation or deletion of JmjC. Another example is the KDM6A variant found in the urothelial cancer cell line T-24 with heterozygous mutations at E895* and E902*. These similar-sized variants are still expressed endogenously and can be detected as a ~ 97 kDa band on WB [18]. It remains speculative whether these fragments display dominant negative effects and are actively involved in generating the cancerous T-24 phenotype. Other variants, like the moderately frequent Q333*, lack both IDR and JmjC and could potentially impair the TPR8. Apart from lost demethylase activity, these variants likely have impaired interactions with other proteins. We have recently shown that KDM6A associates with RBBP5 in urothelial cancer cell lines dependent on the mutation status of KDM6A and KMT2C/D proteins [18] suggesting vital interactions of KDM6A with the COMPASS complex. Here we showed that TPR substitution variants did not affect RBBP5 binding. However, especially the KDM6A mutation D336G has been shown to be predominantly cytoplasmic in HeLa cells presumably due to impaired binding to ASH2L in a pull-down experiment and reduced nuclear import by the KMT2C/D complex [17]. In a previous study we observed that KDM6A nuclear import was strongly decreased after double, but not single, knock down of KMT2C and KMT2D proteins [18]. Systematic deletion of KDM6A domains clearly indicated that all domains, including TPR and IDR, are necessary for binding of RBBP5 (Fig. 3) independent of demethylase activity. Although JmjC alone does not bind RBBP5, the presence of this domain enhanced binding in KDM6A WT compared to KDM6A ΔJmjC and KDM6A TPR. A recently published study indicated that RBBP5, WDR5 and the KDM6A JmjC domain share similar recognition and binding motifs at the Histone 3 tail (aa 1–57) [20]. Furthermore, RBBP5, but not WDR5, bound to H3K27me3 modified peptides and K27F.

All KDM6A variants were located in the nucleoplasm, albeit to different extents, independent of their mutation status. Truncation variants, but not substitution variants, are characterized by eliciting (1) cytoplasmic DNA release, (2) enhanced levels of RAD51 and phospho-γH2AX as indicators of DNA damage, and (3) defects of mitosis caused by missegregated chromosomes at anaphase and persisting chromatin bridges at telophase and cytokinesis (Figs. 2, 5, S4). All observed effects occurred on a short time scale within 36–48 h. While transient and stable overexpression of the KDM6A WT reduces long-term cell growth and colony formation [18], we never observed effects of this kind, neither short-term nor long-term. In general, aneuploidy, replication stress and mitosis errors are common in cancers [21]. Accordingly, all cancer cell lines used in this study exhibit these features, but they are profoundly enhanced after induction of KDM6A truncation variants (Fig. 5). Among the severe phenotypes, cytoplasmic DNA release was most commonly observed. All KDM6A truncation variants were associated (directly or indirectly) with the DNA released from the nucleus as indicated by localization analysis and the cellular back mapping approach. Nuclear DNA release is the presence of cytoplasmic DNA caused by a yet unknown mechanism. We speculate that appearance of cytoplasmic DNA could be caused by (1) pulverized micronuclei or (2) chromosome fragments without envelope or (3) active nuclear release due to impaired nuclear integrity or DNA damage [22]. Our observations point towards mitotic defects (Fig. 5) followed by apoptosis (Fig. 6). However, we cannot rule out additional mechanisms, as we do not have conclusive data on cGAS/STING activation that is expected in response to cytoplasmic DNA accumulation [23]. Moreover, introduction of KDM6A variants, especially truncated ones, elicited elevated phospho-γH2A.X levels (Figure S8). Phospho-γH2A.X is activated during the DNA damage stress response [24]. Enrichment of proteins involved in DNA repair and stress response (DDR) appeared in our MS-data analysis from three different urothelial cancer cell lines with stably or transiently transfected KDM6A WT (Figure S6). Notably, KDM6A activity in differentiating embryonal stem cells has been linked to DNA damage response pathways by colocalization with γH2A.X positive foci [24]. In addition, as an oxygen-dependent enzyme KDM6A serves as a sensor to control chromatin and cell fate [25]. Thus, overexpressed (with a high dose-effect) and impaired KDM6A variants as well as oxygen-related stress have the tendency to increase DNA damage. This phenomenon was also observed in diabetic kidney disease [26]. An additionally prominent feature of truncated KDM6A variants was a high degree of co-localization with NPM1 at extranuclear DNA and they appeared, like all variants, as a mixed population in the nucleoplasm (Fig. 4) as suggested by scatterplot and cellular back mapping analysis. As NPM1 is involved in rRNA processing, ribosome maturation and shuttling of ribosomal subunits between nucleoli, nucleoplasm and cytoplasm [27], KDM6A might be involved in these processes, too. However, the co-occurrence of NPM1 and KDM6A truncation variants may rather result from the role of NPM1 as a chaperone [28]. At this stage, we cannot completely rule out activation of the unfolded protein response pathway (UPR) or ER proteostasis [29] by truncated KDM6A, but consider it rather unlikely for the following reasons. (1) We observed correct nuclear localization of all truncated variants (Figure S4). (2) Perinuclear aggregation was observable in all KDM6A WT, substitution, truncation and control (eGFP) variants. (3) In truncated variants with a severe phenotype, KDM6A protein was always associated with DNA and never freely distributed throughout the cytoplasm. It remains possible that UPR stress sensors contribute to activation of the nuclear DNA damage response [29], which is confirmed by enhanced phospho-γH2A.X levels. As KDM6A itself might act as a critical stress sensor, it is difficult to ascertain at this stage which signaling cascade might explain our observations best.

Our observations hint at possible new functions or involvement of KDM6A in the cell cycle. Specifically, the following questions are raised: First, what is the role of KDM6A during mitosis and to which extent is any such function dependent on its catalytic activity and its interplay with RBBP5 and further components of the KMT2C/D-COMPASS complex? Notably, many lysine demethylases (KDM) have cell cycle specific roles [30, 31]. KDM4C, KDM1A and KDM7B have already been linked to mitosis by regulation of chromosome segregation, transcriptional activation of mitotic checkpoint complex components (see refs in (30)). Moreover, WDR5 and KMT proteins, likely KDM6A interaction partners, have also been shown to be involved in mitosis [32, 33]: WDR5 is part of the midbody in the spindle apparatus [31]. Intriguingly, we found endogenous KDM6A located along the midbody (Figure S8). Secondly, the multifaceted functions of NPM1 in chromatin remodeling, DNA repair, cell cycle control, apoptosis, mitotic spindle, centromere and cytoskeleton binding [28, 34] and its prominent enrichment in MS analysis suggests an important link between both proteins. Thus, under which conditions and in which manner do both proteins directly or indirectly interact?

## Conclusion

Overall, our approach combining biochemical, cellular and imaging techniques revealed that truncating KDM6A mutations lacking TPR, JmjC and/or IDR dramatically increase nuclear damage and apoptosis whereas single substitution variants with diminished demethylase activity or unknown features (T726K) show at most a weak trend towards these effects which are summarized in Fig. 6C. Our findings point towards a pivotal balance between the different domains of KDM6A, which, if disturbed, might facilitate interference of these KDM6A variants with cellular processes mediated by the wildtype protein and its partners.

## Methods

### Cell lines and cell culture

Parental T-24 and SW-1710 urothelial carcinoma cell lines were obtained from the DSMZ (Braunschweig, Germany). Cells were cultured and treated in DMEM GlutaMAX-I (Gibco, Darmstadt, Germany) with 10% fetal bovine serum (FBS; Gibco™, Thermo Fisher Scientific) and 100 U/ml penicillin/100 μg/ml streptomycin (Sigma-Aldrich, Darmstadt, Germany), except for HBLAK cells, which were solely cultured in CnT-Prime Epithelial Culture Medium (CELLnTEC, Bern, Switzerland) without any additives. Cells were incubated at 37 °C in a humidified atmosphere with 5% CO2. STR (short tandem repeat) profiling via DNA fingerprint analysis was performed for all cell lines in this study and is available upon request.

### Generation of eGFP-KDM6A substitution and deletion variants

eGFP-KDM6A wildtype (WT) was synthesized by BioCat (Heidelberg, Germany) by cloning a codon-optimized eGFP-KDM6A (both sequences full-length, KDM6A main isoform 1, Uniprot ID O15550, without additional linker between eGFP and KDM6A) into the pcDNA 3.1(+) vector, using NheI and NotI as flanking restriction sites. Generation of eGFP-KDM6A substitution variants was done by using site-directed mutagenesis (SDM) with 10 ng eGFP-KDM6A WT plasmid and mutagenesis primers (Table S2A). 1.25 U PrimeSTAR GXL DNA Polymerase (Takara Bio, Kusatsu, Shiga, Japan) was used in PCR reactions. Successful PCR amplification and product length was checked by gel electrophoresis. After DpnI (NEB, Ipswich, MA, USA) digestion (20 U for 1 h at 37 °C), SDM amplicons were transformed into *Escherichia Coli* XL10-Gold® (Stratagene, Santa Clara, CA, USA) and spread on LB amp plates. Colonies were picked, grown and DNA was isolated using a QIAprep Spin Miniprep Kit (QIAGEN, Hilden, Germany). After sequencing, DNA from positive KDM6A substitution clones was re-transformed into *E. coli* XL10-Gold® (Stratagene) and purified at a large scale using NucleoBond Xtra Maxi Plus EF kit (Macherey-Nagel, Dueren, Germany). Substitutions were then re-confirmed by sequencing. Generation of eGFP-KDM6A deletion variants was done by “modularized” cloning of three inserts: TPR, res. 1–390, IDR, res. 391–885 and JmjC, res. 886–1401 from the original wildtype eGFP-KDM6A pcDNA3.1(+) were cloned into the pEGFP-C1 vector for the desired combinations. Each restriction enzyme (RE) site produces a two amino acids long linker. Constructs with one insert (eGFP-TPR, eGFP-IDR) have BspEI and HindIII as flanking RE sites. Constructs with two inserts (eGFP-KDM6A ΔTPR, ΔIDR, ΔJmjC) have BspEI and EcoRI as flanking RE sites and HindIII as middle RE site. The control construct eGFP-KDM6A_cloning_ has BspEI and KpnI as flanking RE sites and HindIII and EcoRI as mid RE sites. Amplification primer (Table S2A) were designed according to the desired combination with appropriate overhangs and synthesized by Eurofins Genomics (Ebersberg, Germany). T4 DNA ligase (NEB) was used for insert ligation (10 min at RT, 3.1 insert:vector ratio). NucleoSpin Gel and PCR Clean-Up (Macherey-Nagel) was used to extract and clean up DNA. Cloning products were transformed into *Escherichia Coli* XL10-Gold® (Stratagene). An appropriate number of colonies were picked, grown and the DNA was isolated using a QIAprep Spin Miniprep Kit (QIAGEN). After sequencing, positively cloned DNA was re-transformed into *E. coli* XL10-Gold® (Stratagene) and purified in a large scale using a NucleoBond Xtra Maxi Plus EF kit (Macherey-Nagel).

### Transient transfection

For transient transfection, cells were seeded into 6-well plates with glass cover slips (for imaging) or without (for activity, western blot). 24 h later, cells were transfected at ~ 90% confluence using X-tremeGENE™ 9 or HP (Roche, Basel, Switzerland; application dependent use) in a 2:1 ratio (v/w) of transfection reagent to DNA. Total DNA transfected per well (9.6 cm^2^) did not exceed 2 μg. Transfection was carried out for 24–48 h for activity assay and Western Blot applications and 36 h for localization analysis.

### Cell death analysis by flow cytometry

One hundred fifty thousand cells (SW-1710) and two hundred thousand cells (T-24) per well of a six well plate were seeded and reversely transfected with XtremeGENE™ HP (Roche). After 16 h cells were split into two wells. 48 h post transfection, supernatant and cells were collected, centrifuged at 1000 rpm for 5 min, washed with ice-cold 1x Annexin binding buffer (Serva, Heidelberg, Germany) and centrifuged again. The pellet was resuspended in 75 μl 1x Annexin binding buffer containing 4.5 μl Annexin V-APC (Serva) and 7.5 μl PI (1 mg/ml, Serva) and incubated for 15 min in the dark at RT. The suspension was diluted with 500 μl 1x Annexin binding buffer, centrifuged, washed and fixed with 0.5% methanol-free formaldehyde for 20 min on ice. The reaction was stopped with 500 μl 1x Annexin binding buffer. FACS measurements and analysis was performed using the MACSQuant Analyzer X and MACSQuantify Software (Miltenyi Biotec, Bergisch-Gladbach, Germany). In total, 50,000 cells/experiment were analyzed in three independent experiments.

### Co-IP and Western blot analysis

An appropriate amount of cells were lysed by suspension in SDS-free RIPA like buffer (RLB) consisting of 50 mM Tris-HCl (pH 7.5), 0.3% CHAPS, 150 mM sodium chloride, 1 mM sodium vanadate (Na_2_VO_4_), 10 mM sodium fluoride (NaF), 1 mM ethylene diaminetetraacetate (EDTA), 1 mM ethylene glycol-bis(β-aminoethyl ether)-N,N,N′,N′,-tetraacetate (EGTA), 2.5 mM tetrasodium pyrophosphate Na_4_O_7_P_2_. Dithiothreitol and 1x HALT™ protease inhibitor cocktail (Sigma-Aldrich) were freshly added. Lysis was followed by immediate freezing in liquid nitrogen, thawing on ice for 30 min and repeated mixing by pipetting for 30 s each. The lysate was either directly separated by SDS-PAGE on a 4–20% gradient gel or used in the following Co-IP steps. For Co-IP, the GFP containing lysate was incubated with GFP-trap dynabeads (Chromotek, Planegg-Martinsried, Germany) for 1 h at 4 °C with constant mild agitation to pull-down eGFP-KDM6A variants and complexed proteins. Dynabeads were magnetically separated and washed three times with Co-IP buffer, resuspended in SDS-PAGE loading buffer, boiled and separated on a 4–20% gradient gel. The gel was transferred onto an methanol activated PVDF-membrane. The membrane was blocked in TBS/0.1% Tween (TBS-T) and 5% BSA for 1 h at RT and subsequently incubated with the respective primary antibody (Table S2B) in TBS-T, 1% BSA overnight at 4 °C. The membrane was washed three times in TBS-T at RT. The secondary antibody (Table S2B) was applied for 1 h at RT in TBS-T, 1% BSA. The membrane was washed again three times and Clarity™ ECL (Bio-Rad, Hercules, CA, USA) was used to develop the signal with the ChemiDoc Imaging System (Bio-Rad, USA).

### ELISA-based demethylase activity assay

Preparation and lysis of the cells was done the same way as described for Co-IP. However, before the GFP-trap dynabeads were added, the fluorescent emission signal of the lysate was measured (470 nm ex., 485–650 nm em.) in a microliter cuvette. The dynabeads were then mixed in the lysate for 1 h at 4 °C under constant, mild agitation. Beads were then magnetically separated, the fluorescent emission of the remaining lysate was measured again with the same specifications as above. The delta in the emission spectra before and after bead incubation in the range of 500–530 nm was used to calculate the amount of eGFP-KDM6A pulled out of the lysate in each run. To eliminate residual RLB buffer, beads were washed two times with the activity assay (AA) buffer (50 mM TRIS-HCl (pH 7.45), 0.02% Triton X-100, 100 μM α-ketoglutarate, 50 μM Fe(NH_4_)_2_(SO4)_2_*6 H_2_O, 100 μM ascorbic acid, 1 mM TCEP, cofactors and TCEP being added freshly to avoid oxidation. H3K27me3 (ProteoGenix, Schiltigheim, France) and H3K27me2 (BioCat) peptides (Table S2C) were dissolved in AA buffer and mixed with the loaded beads. The beads were incubated with the peptides for 4 h at 30 °C while maintaining constant suspension. Afterwards, the beads were magnetically separated and the supernatant containing the biotin-labeled peptide was loaded onto a streptavidin-coated 96-well plate (triplicate per variant/control, 50 μl per well). After 1 h of biotin binding at RT and removal of the solution, the wells were loaded with 50 μl of the a-H3K27me2 antibody in 0.1% TBS-T and incubated for 1 h at RT while shaking gently. The wells were then washed three times with 150 μl TBS-T. Subsequently, 50 μl of a 1:1000 alkaline phosphatase (ALP)-conjugated secondary antibody was added and incubated for 30 min at RT. The wells were washed four times for 5 min. For detection, 100 μl of p-nitrophenyl phosphate (pNPP, Sigma-Aldrich) was added into each well and incubated for 10 min at RT in the dark, mixing thoroughly. The reaction was then quenched by 100 μl 1 M NaOH. The signal was measured in a plate reader at 405 nm absorption, quantified, normalized and fitted. To fit the ELISA readout, we used a 4-PL-regression normalized to the standard curve (Figure S2). The WT fit overlaid with the standard curve was used to directly calculate the relation between the amount of fluorescence signal and demethylated product. WT 4PL-regression fit was applied to calculate c_50_ as a reference point to compare the WT value with the variants. Additionally, eGFP reference measurements were used to calculate the absolute amount of protein input and calculate a specific activity. Initial validation for the assay was done with recombinant full-length KDM6A (Active Motif, Carlsbad, CA, USA).

### Immunocytochemistry

Depending on the antibody requirements two protocols were used. For ICC with primary antibody incubation overnight, cells were seeded on coverslips, transiently transfected and fixed at > 80% confluence with 1% (v/v) para-formaldehyde, 0.02% (v/v) Triton X-100 for 20 min at RT. Blocking and permeabilization was done with 1% (w/v) BSA, 0.1% (w/v) saponin in PBS for 30 min at RT. After overnight night incubation at 4 °C, coverslips were washed with PBS and incubated with the secondary antibody for 1 h at RT. Slides were washed with PBS, stained with DAPI, washed again frequently with PBS and mounted. For ICC with primary antibody incubation for 1 h RT, cells were prepared as before, but fixed with 4% FA (v/v) for 10 min at RT. Permeabilization was done with 0.5% (v/v) Triton X-100 for 3 min at RT and blocking with 1% (w/v) BSA in PBS for 30 min at RT. The primary antibody was incubated with shaking for 1 h at RT, coverslips were washed with PBS and incubated with shaking with the secondary antibody for 1 h at RT. Slides were washed with PBS, stained with DAPI, washed again several times with PBS and mounted. Primary and secondary antibodies are listed in Table S2.

### Microscopy and image processing

Confocal imaging with live or fixed cells was performed on a confocal laser scanning microscope FV1000 IX81 inverted microscope (Olympus, Shinjuku, Japan) using a 60x water immersion UPLSAPO NA 1.2 objective. DAPI, eGFP and Star Red were excited at 405 nm, 488 nm and 635 nm, respectively, with the internal FV10-MARAD-2 main laser unit. Star Orange was excited at 559 nm with an external Opti λ 559 diode laser (NTT Electronics, Yokohama, Japan). Internal PMT detectors (Olympus) were used for detection. Confocal laser scanning microscope (LSM) processing routine was carried out with the freely accessible Fiji and Huygens Pro 20.10 (SVI, Hilversum, Netherlands) for deconvolution. For deconvolution of images fulfilling the Nyquist criterion, we used an automatically computed theoretical point spread function based on our known microscopic parameters and a model of the Olympus IX81 provided by SVI and performed 30 iterative steps of classic maximum likelihood estimation (CMLE) on our images. The signal-to-noise ratio was determined for each channel and then kept constant during image processing.

### Scatterplot generation and cellular back mapping

We used the freely available, open source Fiji plugin ScatterJ [19]. Processed single slice images were converted into 8-bit grey scale tagged image file formats (.tiff) and opened in ScatterJ. 256 × 256 pixel scatterplots were saved as xy-lists (.dat) for processing in OriginPro. Within scatterplot, regions were selected using Fiji free-hand-tool and back mapped to the original image. The back-mapped image, as well as channel-wise images, were saved as text sequences (.dat) or portable networks graphics (.png) for further image and matrix analysis in OriginPro. The resulting cellular back-mapping-images representing the pixel-wise analysis of defined scatterplot populations are shown in Fig. 4 and the corresponding Figure S7.

## Supplementary Information

**Additional file 1: Table S1.** Predicted solubility and pI of KDM6A substitution and truncation variants. Predicted solubility and pI of KDM6A substitution and truncation variants using Protein-Sol from the University of Manchester based on published sequences [35, 36]. Compared to KDM6A WT protein TPR and ΔIDR show reduced solubility, whereas the IDR sequence alone is predicted to be most soluble. Single substitution do not affect pI and solubility, as expected. Among nuclear proteins, such as components of the COMPASS complex (KMT2C/D, WDR5, RBBP5) and NPM1, KDM6A is the least soluble. Protein identifier were taken from UniProt.

**Additional file 2: Table S2.** Primer, antibodies and peptides used in this study. **A.** Primer used for site-directed mutagenesis and amplification. **B.** Primary and secondary antibodies used in this study. **C.** H3K27-peptides used in this study.

**Additional file 3: Table S3.** *P*-Values and scoring results from Figs. 5 and 6**. A.** P-Values from Fig. 5. **B**. Scoring results used in Fig. 5. **C**. P-Values from Fig. 6.

**Additional file 4: Figure S1.** Bioinformatic analysis of KDM6A tetratricopeptide repeats. **A.** Alignment of annotated KDM6A TPRs using the TPRprediction and alignment tool published in [37]. All annotated KDM6A TPRs with the given amino acid range (see label) were compared for their TPR conservation to the consensus sequence as published in [38] with the most conserved amino acids W4, L7, G8, Y11, A20, Y24, A27 and P30. Identity and *p*-values are shown on the right site. The TPR motif could not be identified for TPR3 (aa 170–199). Color coding according to CLUSTAL W alignment. **B.** Secondary structure prediction of TPR3 using the Quick2D on the MPI Bioinformatics toolkit server [39, 40] indicated a high probability for aa 14–26 to form an α-helix, but aa 1–10 tend to form either an α-helix or a β-strand. Of note, the canonical aa 34 TPR repeating unit forms a helix-turn-helix motif [38]. In general, the N-terminal TPRs 1–4 show less strict conservation towards the canonical TPR sequence compared to the C-terminal TPRs 6–8.

**Additional file 5: Figure S2.** Activity assay and data analysis. In this section, we describe our data analysis workflow. ***A.*** *delta* (grey shaded area) in fluorescence emission spectrum of eGFP-KDM6A WT lysate before incubation and after incubation with GFP-trap dynabeads. For data analysis, the amount of pulled fluorescent protein was determined by subtracting the eGFP fluorescence emission (470 nm excitation) after incubation with GFP-trap dynabeads from eGFP fluorescence emission before incubation with eGFP-trap dynabeads. Fluorescence intensities in the range of 500–530 nm were integrated subsequently (later referred to as integrated fluorescence). **B.** Maximum-normalized spectra obtained from crude cell lysate of eGFP, eGFP-KDM6A WT or eGFP-KDM6A Q1133A samples. All fluorescence emission spectra of the substitution variants had a similar profile between 500 and 530 nm. This was used to compare the amount of protein for each variant. Q1133A variant is shown exemplarily for all other substitution variants. **C.** Fluorescence emission spectra of WT and ΔJmjC. The high signal of ΔJmjC towards the blue end of the spectrum is due to scatter, which could indicate protein instability under the given (low salt) buffer conditions. **D.** 1–238 ng demethylated product (H3K27me2 peptide, MW = 2945.5 g/mol) was obtained from at least nine data points from three independent experiments, each in triplicates and fitted with a four parameters logistic regression (4-PL) according to Eq. 1: $\mathrm{y}=\mathrm{d}+\frac{\mathrm{a}-\mathrm{d}}{1+{\left(\frac{\mathrm{x}}{\mathrm{c}}\right)}^{\mathrm{b}}}$ (1) with y = assay readout absorption, x = amount of product in A., amount of KDM6A species, either by fluorescence in B. or amount of protein in D., a = background signal, d = maximum signal, b = slope factor, c = c50, x-value at half maximum y.The following fit results were obtained: *a = 0.29, b = 1.27, d = 1.73 and c = 62.4 ng*. **E.** The amount of protein was plotted against absorption (shown for KDM6A WT) and fitted accordingly with 4-PL, with *c* being the only open parameter. c50 for WT was determined with 518,000 +/− 56,000 AU. To correct for background and maximum signal fluctuation between experiments, they were normalized to the values obtained from the product curve fit (*a = 0.29, d = 1.73*). **F.** A defined amount of recombinant eGFP (27 kDa) was used to convert the x-axis from fluorescent signal to the amount of protein. The linear dependency was used to calculate the amount of eGFP-KDM6A (181 kDa) per fluorescent unit. The molar integrated fluorescence (500–530 nm) from emission spectra for recombinant eGFP (MW = 26.9 kDa) was determined as 2.34E+ 18 AU/mol from the relation 1 ng eGFP = 86,900 [AU]. Therefore, 1 ng eGFP-KDM6A WT would yield an integrated fluorescence signal of 1.29E+ 03 AU. **G.** Data from **E.** and **F.** were used to transform the x-Axis with the fluorescent signal into the amount of KDM6A protein,. Approximated from **D.,** the respective c50 always corresponded to 62.4 product. This correlation was used to calculate the specific activity for each variant. Given the fixed time of 240 min per assay, the specific activity under these conditions was calculated. The c50 for all available substitution variants was obtained in the same manner, fixing all parameters except c, using it as a relative measure of activity. At least four independent triplicate measurements were obtained for each substitution variant. **H.** absorption for truncated variants, three repeats with triplicates each. Since the truncated variants showed an unusual spectrum with a high scatter fraction at lower wavelengths (see **C.**), a quantification was impossible. Therefore, we qualitatively confirmed, that all truncated variants with a JmjC domain are catalytically active (green boxes) and those without JmjC domain are catalytically dead (red boxes). The distribution of the signal over the background is an evidence for activity in all truncated variants with a JmjC domain. ΔJmjC and TPR do not exceed assay background levels (zero), while WT, ΔIDR, ΔTPR and JmjC are all above background. **I.** Activity of T726K is dependent on post-expression time. WT activity does not change significantly between 24 h (black) to 48 h (grey), whereas in T726K activity is slightly lower than WT after 24 h (blue) and strongly reduced after 48 h (cyan) when comparing c50-values.

**Additional file 6: Figure S3.** Control experiments for antibody staining specificity and endogenous KDM6A protein levels. SW-1710 and T-24 cells were transfected with KDM6A WT or ΔIDR variants and stained with two different antibodies (sc-514,859, Santa Cruz biotechnology and CST-33510, Cell Signaling) raised against epitopes within the KDM6A central region (IDR) and 2nd antibody labeled with AbStar Red (Abberior). Clearly, both antibodies (in red channel) detect transfected KDM6A WT protein (very strong signals in green channel), but not the ΔIDR variant. The untransfected control cells show endogenous KDM6A in both cell lines stained with both KDM6A antibodies.

**Additional file 7: Figure S4.** Localization study of KDM6A variants in normal and invasive urothelial (cancer) cells. **A.** KDM6A substitution and deletion variants are localized in the cytoplasm and nucleus in T-24 cells. Substitutions cause mild effects, whereas deletion variants cause stronger cellular responses such as DNA release and mitotic errors. **B.** Representative KDM6A deletion variants are localized in the cytoplasm and nucleus in HBLAK. Two different phenotypes have been observed: a milder phenotype (majority of cells) and a phenotype (minority of cells) which reproducibly causes stronger cellular responses such as DNA release and mitotic errors. DAPI counterstaining of the nucleus and the respective overlay of both channels (DAPI in blue and GFP in green) is displayed. Images are taken in 2048 × 2048 resolution using a widefield microscope Olympus FX81 with 60x oil objective with post-processing using HuygensPro20.10. Effects of KDM6A variants in SW-1710 cells is shown in Fig. 2.

**Additional file 8: Figure S5.** Western Blot control experiments and raw blots. **A.** eGFP-KDM6A TPR whole cell lysate and the corresponding IP fraction was separated on a Western Blot and stained with eGFP (CST) or RBBP5 (CST) antibodies. RBBP5 precipitated with eGFP-KDM6A TPR. B. Dynabeads washing control on same membrane, washed with 3x RIPA like buffer (RLB). While KDM6A WT pulls a clearly visible RBBP5 band and a low WDR5 band, both are undetectable in eGFP-transfected cells alone (negative control). **C.** Raw blots from Fig. 3. Lysate and Co-IP for selected KDM6A variants and eGFP. IP and corresponding lysate lanes were also on the same blot. All experimental conditions for the blots presented were kept constant. **D**. Raw blots from Fig. 1B**/D**. Protein lanes shown in main figures are in red boxes. The PageRuler Plus Prestained protein ladder (Thermo Fisher) was used for orientation.

**Additional file 9: Figure S6.** Pathway enrichment analysis of MS data from KDM6A-tagGFP2 cell lines. **A.** Data from quadruplicates of three independent experiments of two different KDM6A-tagGFP2 cell lines using the reactome database. MS data sets were applied to the reactome analysis database. The resulting output shows significantly enriched pathways. The five most interesting pathways are depicted: cell cycle, chromatin organization, DNA repair, RNA metabolism including rRNA processing and protein metabolism including ribosome biogenesis and post-translational modification of histones. **B**. Top ten proteins identified by MS in KDM6A-tagGFP2 stable cell lines VM-CUB1 and RT-112. Among the top ten proteins in KDM6A pull down and subsequent MS, Nucleophosmin (NPM1), Nucleolin, ribosomal subunits 40S and 60S, as well as histone variants, are detectable. Mutational KDM6A and KMT2C/D status and experiments with RT-112 and VM-CUB1 have been recently published [18].

**Additional file 10: Figure S7.** Cellular back mapping identifies co-localizing protein populations at extranuclear DNA segments. **A**. images shown in Fig. 4, all channels. **B**. Analysis of KDM6A TPR and NPM1: Cellular back mapping identifies a colocalizing population (orange) and free KDM6A TPR (green) and NPM1 (red) at cytoplasmic DNA segments. **C**. scatterplot analysis and cellular back mapping of KDM6A variants and DAPI as indicator of KDM6A-DNA complexes. In KDM6A WT scatterplot we identified four populations: 1 = cytoplasmic KDM6A (without DAPI) and 4 = DAPI (without KDM6A), as well as, populations #2 and #3, which resemble different ratios of KDM6A-DNA complexes. Population 2 is located at the lamina and in nucleolar puncta, and population 3 is located within the nucleoplasm. Substitution variant T726K shows minor differences in populations #2 and #4, as the lamina in #2 is less clearly visible, but strongly enriched in population #4. Contour scatterplot of JmjC or TPR with DAPI identified only three populations, with population #1 being absent. Back mapping clearly indicates compartment-wise localization of different DAPI-KDM6A JmjC or TPR compositions.

**Additional file 11: Figure S8.** Distribution and expression levels of p-γH2A.X and RAD51 after KDM6A overexpression and endogenous localization of KDM6A during mitosis. **A**. p-γH2A.x protein level in untransfected and KDM6A variants transfected T-24 cells. Note, that even in strongly overexpressed KDM6A WT cells, p-γH2A.X was not increased, but strongly so in KDM6A JmjC and ΔTPR cells. **B**. Endogenous localization of KDM6A (CST antibody, 2nd antibody goat-anti-rabbit AbStar red) as imaged with Leica STED SP8 in HBLAK cells. For STED, the sample was depleted with 35% of 775 nm laser. **C**. RAD51 proteins were detected at a persisting chromatin bridge of two daughter cells, with cell 1 being KDM6A ΔTPR positive. Image was taken with Abberior STED, excitation laser 640 nm 5% and depletion laser 775 nm with 40%.

## Data Availability

All data generated or analysed during this study are included in this published article and in supplementary information files or are available from the corresponding authors on reasonable request.

## Abbreviations

4-PL: Four parameter logistic
aa: Amino acid
AA: Activity assay
CMLE: Classic maximum likelihood estimation
COMPASS: Complex proteins associated with Set1
DDR: DNA repair and stress response
ELISA: Enzyme-linked immunosorbent assay
FACS: Fluorescence Activated Cell Sorting
GO: Gene ontology
IDR: Intrinsically disordered protein
JmjC: Jumonji C domain
KDM: Lysine demethylase
KMT: Lysine methyltransferase
M: Manders coefficient
MS: Mass spectrometry
NPM: Nucleophosmin
P: Pearson coefficient
PTM: Post-translational modification
RBBP: Retinoblastoma-binding protein
RLB: RIPA-like buffer
SDM: Site-directed mutagenesis
SWI/SNF: SWitch/Sucrose non-fermentable
TPR: Tetratricopeptide repeat
UTX: Ubiquitously transcribed tetratricopeptide repeat, X chromosome
WDR: WD-repeat containing protein
WRAD: WDR5-RBBP5-ASH2L-DPY30 complex
WT: Wildtype
