# Supplementary material for: KDM6A mutations promote acute cytoplasmic DNA release, DNA damage response and mitosis defects
Source: BMC Mol Cell Biol. 2021 Oct 26;22:54. doi: 10.1186/s12860-021-00394-2 (PMC8549169; doi:10.1186/s12860-021-00394-2)
Supplement: Supplementary file 1 — Additional file 1: Table S1. Predicted solubility and pI of KDM6A substitution and truncation variants. Predicted solubility and pI of KDM6A substitution and truncation variants using Protein-Sol from the University of Manchester based on published sequences [35, 36]. Compared to KDM6A WT protein TPR and ΔIDR show reduced solubility, whereas the IDR sequence alone is predicted to be most soluble. Single substitution do not affect pI and solubility, as expected. Among nuclear proteins, such as components of the COMPASS complex (KMT2C/D, WDR5, RBBP5) and NPM1, KDM6A is the least soluble. Protein identifier were taken from UniProt. [file 12860_2021_394_MOESM1_ESM.docx]

**Table S1**: **Predicted solubility and pI of KDM6A substitution and truncation variants.**

| **KDM6A variant** | **pI** | **Predicted solubility** | **Protein identifier** |
| --- | --- | --- | --- |
| ΔIDR | 6.98 | 0.167 | [O15550](https://www.uniprot.org/uniprot/O15550) |
| TPR | 6.10 | 0.209 |  |
| JmjC | 9.06 | 0.266 |  |
| **KDM6A WT (UTX)** | **7.68** | **0.278** |  |
| ΔTPR | 7.12 | 0.336 |  |
| ΔJmjC | 9.23 | 0.343 |  |
| IDR | 9.62 | 0.483 |  |
| T726K | 7.88 | 0.278 |  |
| T726V | 7.68 | 0.277 |  |
| **Nuclear proteins** | **pI** | **Predicted solubility** | **Protein identifier** |
| NPM1 | 4.61 | 0.866 | [P06748](https://www.uniprot.org/uniprot/P06748) |
| KMT2C | 6.33 | 0.412 | [Q8NEZ4](https://www.uniprot.org/uniprot/Q8NEZ4) |
| KMT2D | 5.47 | 0.498 | [O14686](https://www.uniprot.org/uniprot/O14686) |
| WDR5 | 9.4 | 0.396 | [P61964](https://www.uniprot.org/uniprot/P61964) |
| RBBP5 | 4.94 | 0.654 | [Q15291](https://www.uniprot.org/uniprot/Q15291) |

Predicted solubility and pI of KDM6A substitution and truncation variants using Protein-Sol from the University of Manchester based on published sequences [1, 2]. Compared to KDM6A WT protein TPR and ΔIDR show reduced solubility, whereas the IDR sequence alone is predicted to be most soluble. Single substitution do not affect pI and solubility, as expected. Among nuclear proteins, such as components of the COMPASS complex (KMT2C/D, WDR5, RBBP5) and NPM1, KDM6A is the least soluble. Protein identifier were taken from UniProt.

1. Bateman, A., et al., *UniProt: a worldwide hub of protein knowledge.* Nucleic Acids Research, 2019. **47**(D1): p. D506-D515.

2. Hebditch, M., et al., *Protein-Sol: a web tool for predicting protein solubility from sequence.* Bioinformatics, 2017. **33**(19): p. 3098-3100.
