# Supplementary material for: KDM6A mutations promote acute cytoplasmic DNA release, DNA damage response and mitosis defects
Source: BMC Mol Cell Biol. 2021 Oct 26;22:54. doi: 10.1186/s12860-021-00394-2 (PMC8549169; doi:10.1186/s12860-021-00394-2)
Supplement: Supplementary file 2 — Additional file 2: Table S2. Primer, antibodies and peptides used in this study. A. Primer used for site-directed mutagenesis and amplification. B. Primary and secondary antibodies used in this study. C. H3K27-peptides used in this study. [file 12860_2021_394_MOESM2_ESM.docx]

**Primer, antibodies and peptides used in this study**

**Table S2A: primer used for site-directed mutagenesis and amplification**

| **variant/direction** | **Sequence 5’-3’** | |
| --- | --- | --- |
| E315Q Fwd | ATCGACAAGAGCCAGGCCTC | |
| E315Q Rev | AGGCCTGGCTCTTGTCGATG | |
| D336G Fwd | ATGGGCGCCCTGCAGGCCTACAT | |
| D336G Rev | ATGTAGGCCTGCAGGGCGCCCAT | |
| T726K Fwd | CAACATCCTGAAGGTGCCTGAGAC | |
| T726K Rev | GTCTCAGGCACCTTCAGGATGTTG | |
| T726V Fwd | CAACATCCTGGTCGTGCCTGAGAC | |
| T726V Rev | GTCTCAGGCACGACCAGGATGTTG | |
| P966R Fwd | CAAATCCCAATAATCGTGTGACCGTGATC | |
| P966R Rev | GATCACGGTCACACGATTATTGGGATTTG | |
| Q1133A Fwd | CATGAATACAGTGGCGCTGTACATGAAG | |
| Q1133A Rev | CTTCATGTACAGCGCCACTGTATTCATG | |
| H1329A Fwd | GAGGAGCCTGCCGCCTACTGTAGCATCTG | |
| H1329A Rev | CAGATGCTACAGTAGGCGGCAGGCTCCTC | |
| V1338F Fwd | AGGTGGAGTTCTTCGACCTGCTGTT | |
| V1338F Rev | AACAGCAGGTCGAAGAACTCCACCT | |
| C1361Y Fwd | ACTGCCAGGACTACGCCAGAAAGA | |
| C1361Y Rev | TCTTTCTGGCGTAGTCCTGGCAGT | |
| **domain/direction** | **RE** | **Sequence 5’-3’** |
| TPR front Fwd | BspEI | ATATTCCGGAATGAAGTCCTGCGGCGTGT |
| TPR front Rev | HindIII | TATAAGCTTCAGGGCGGATGTATTGCTGCA |
| TPR front stop Rev | HindIII | TATAAGCTTTCACAGGGCGGATGTATTGCTGCA |
| IDR front Fwd | BspEI | ATATTCCGGAGCCGCCAGGATCAAGTACCT |
| IDR front Rev | HindIII | TATAAGCTTCATGCTGGGGATGATCTGAGGG |
| IDR back Fwd | HindIII | TATAAGCTTGCCGCCAGGATCAAGTACCT |
| IDR back Rev | EcoRI | ATATGAATTCCATGCTGGGGATGATCTGAGGG |
| IDR back stop Rev | EcoRI | ATATGAATTCTCACATGCTGGGGATGATCTGAGGG |
| JmjC front Fwd | BspEI | ATATTCCGGATCCGTGTCCATCTACCCCTCCTCC |
| JmjC front Rev | HindIII | TATAAGCTTGCTGGAGGCGCTAGGCAGA |
| JmjC front stop Rev | HindIII | TATAAGCTTTCAGCTGGAGGCGCTAGGCA |
| JmjC back Fwd | HindIII | TATAAGCTTTCCGTGTCCATCTACCCCTCCT |
| JmjC back rev | EcoRI | ATATGAATTCGCTGGAGGCGCTAGGCAGA |
| JmjC back stop Rev | EcoRI | ATATGAATTCTCAGCTGGAGGCGCTAGGCA |
| JmjC third Fwd | EcoRI | ATATGAATTCTCCGTGTCCATCTACCCCTCCT |
| JmjC third Rev | KpnI | TATGGTACCGCTGGAGGCGCTAGGCAGA |
| JmjC third stop rev | KpnI | TATGGTACCTCAGCTGGAGGCGCTAGGCA |

**Table S2B: Primary and secondary antibodies**

| **Primary antibodies** | | | | |
| --- | --- | --- | --- | --- |
| **Antibody** | **Source/Isotype** | **Clone** | **Company** | **Cat.-Nr.** |
| α-KDM6A | Rabbit IgG | D3Q1I | Cell Signaling Technology (CST) | 33510 |
| α-KDM6A | Mouse IgG | E-8 | Santa Cruz Biotechnology (SC) | Sc-514859 |
| α-RBBP5 | Rabbit IgG | D3I6P | Cell Signaling Technology | 13171 |
| α-GFP | Mouse IgG2a | 4B10 | Cell Signaling Technology | 2955 |
| α-p-γH2AX | Rabbit IgG | 20E3 | Cell Signaling Technology | 9718 |
| α-NPM1 | Mouse IgG1 | E-3 | Santa Cruz Biotechnology (SC) | sc-271737 |
| α-α-Tubulin | Mouse IgG1 | B-5-1-2 | Sigma-Aldrich | T6074 |
| α-H3K27me2 | Mouse IgG2a | 0324 | Active Motif (AM) | 61435 |
| **Secondary antibodies** | | | | |
| **Conjugation** | **Antigen** | **Host** | **Company** | **Cat.-Nr.** |
| HRP | Rabbit IgG | Goat | Cell Signaling Technology | 7076 |
| HRP | Mouse IgG | Horse | Cell Signaling Technology | 7074 |
| Star Orange | Mouse IgG | Goat | Abberior | STORANGE-1001 |
| Star Red | Rabbit IgG | Goat | Abberior | STRED-1002 |
| ALP | Mouse IgG2a | Rabbit | Sigma-Aldrich | SAB3701179 |

**Table S2C: H3K27-peptides**

| **Peptide** | **Residues** | **Sequence** | **Modification** | **MW** | **Com-pany** | **Cat. Nr.** |
| --- | --- | --- | --- | --- | --- | --- |
| H3K27me3 | 15-34 | Ac-APRKQATKAAR  K(me3)SAPATGG-PEG-Bio | N-Acetyl  C-PEGylate/  Biotin | 2477.9 g/mol | Proteo-  Genix | Custom  order |
| H3K27me2 | 21-44 | NH_2_-ATKAARK(me2)  SAPATGGVKKPH  RYRPG-PEG-Bio | C-PEGylate/  Biotin | 2945.5 g/mol | Epi-Gentek | R-1033 |
