# Supplementary material for: KDM6A mutations promote acute cytoplasmic DNA release, DNA damage response and mitosis defects
Source: BMC Mol Cell Biol. 2021 Oct 26;22:54. doi: 10.1186/s12860-021-00394-2 (PMC8549169; doi:10.1186/s12860-021-00394-2)
Supplement: Supplementary file 3 — Additional file 3: Table S3. P-Values and scoring results from Figs. 5 and 6. A. P-Values from Fig. 5. B. Scoring results used in Fig. 5. C. P-Values from Fig. 6. [file 12860_2021_394_MOESM3_ESM.docx]

**P-Values from Fig. 5 and 6**

**Table S3A: P-Values from Fig. 5**

| Hypothesis tested: | P-value: | Sign.: | P-value: | Sign.: |
| --- | --- | --- | --- | --- |
|  | T-24 | | SW-1710 | |
| Different percentage of total (mono/bi-nuclear, mitosis) normal cells in WT vs. eGFP | 0.9883 | N.S. | 0.0144 | * |
| Different percentage of total (mono/bi-nuclear, mitosis) normal cells in TPR vs. eGFP | 0.0026 | ** | 0.0001 | *** |
| Different percentage of total (mono/bi-nuclear, mitosis) normal cells in JmjC vs. eGFP | 0.0013 | ** | 0.0047 | ** |
| Different percentage of bi-nuclear cells in WT vs. eGFP | 0.2795 | N.S. | 0.5566 | N.S. |
| Different percentage of bi-nuclear cells in TPR vs. eGFP | 0.0164 | * | 0.1608 | N.S. |
| Different percentage of bi-nuclear cells in JmjC vs. eGFP | 0.3400 | N.S. | 0.2134 | N.S. |
| Different percentage of cells with micronuclei (MN) in WT vs. eGFP | 0.4494 | N.S. | 0.7903 | N.S. |
| Different percentage of cells with micronuclei (MN) in TPR vs. eGFP | 0.3132 | N.S. | 0.7956 | N.S. |
| Different percentage of cells with micronuclei (MN) in JmjC vs. eGFP | 0.3192 | N.S. | 0.6114 | N.S. |
| Different percentage of mitotic cells in WT vs. eGFP | 0.7356 | N.S. | 0.6552 | N.S. |
| Different percentage of mitotic cells in TPR vs. eGFP | 0.2191 | N.S. | 0.2182 | N.S. |
| Different percentage of mitotic cells in JmjC vs. eGFP | 0.3425 | N.S. | 0.1106 | N.S. |
| Different percentage of mono-nuclear normal cells in WT vs. eGFP | 0.6352 | N.S. | 0.0803 | N.S. |
| Different percentage of mono-nuclear normal cells in TPR vs. eGFP | 0.0025 | ** | 0.0002 | *** |
| Different percentage of mono-nuclear normal cells in JmjC vs. eGFP | 0.0007 | *** | 0.0017 | ** |
| Different percentage of bi-nuclear damaged cells in WT vs. eGFP | 0.7901 | N.S. | 0.0597 | N.S. |
| Different percentage of bi-nuclear damaged cells in TPR vs. eGFP | 0.0015 | ** | 0.0018 | ** |
| Different percentage of bi-nuclear damaged cells in JmjC vs. eGFP | 0.0237 | * | 0.0232 | * |

**Table S3B: P-Values from Fig. 6**

| Hypothesis tested: | P-value: | Sign.: | P-value: | Sign.: |
| --- | --- | --- | --- | --- |
|  | T-24 | | SW-1710 | |
| Different percentage of viable cells in WT vs. eGFP | 0.0655 | N.S. | 0.0758 | N.S. |
| Different percentage of viable cells in TPR vs. eGFP | 0.0002 | *** | 0.0001 | *** |
| Different percentage of viable cells in JmjC vs. eGFP | 0.0009 | *** | 0.0213 | * |
