# Supplementary material for: KDM6A mutations promote acute cytoplasmic DNA release, DNA damage response and mitosis defects
Source: BMC Mol Cell Biol. 2021 Oct 26;22:54. doi: 10.1186/s12860-021-00394-2 (PMC8549169; doi:10.1186/s12860-021-00394-2)
Supplement: Supplementary file 4 — Additional file 4: Figure S1. Bioinformatic analysis of KDM6A tetratricopeptide repeats. A. Alignment of annotated KDM6A TPRs using the TPRprediction and alignment tool published in [37]. All annotated KDM6A TPRs with the given amino acid range (see label) were compared for their TPR conservation to the consensus sequence as published in [38] with the most conserved amino acids W4, L7, G8, Y11, A20, Y24, A27 and P30. Identity and p-values are shown on the right site. The TPR motif could not be identified for TPR3 (aa 170–199). Color coding according to CLUSTAL W alignment. B. Secondary structure prediction of TPR3 using the Quick2D on the MPI Bioinformatics toolkit server [39, 40] indicated a high probability for aa 14–26 to form an α-helix, but aa 1–10 tend to form either an α-helix or a β-strand. Of note, the canonical aa 34 TPR repeating unit forms a helix-turn-helix motif [38]. In general, the N-terminal TPRs 1–4 show less strict conservation towards the canonical TPR sequence compared to the C-terminal TPRs 6–8. [file 12860_2021_394_MOESM4_ESM.docx]

**Figure S1**


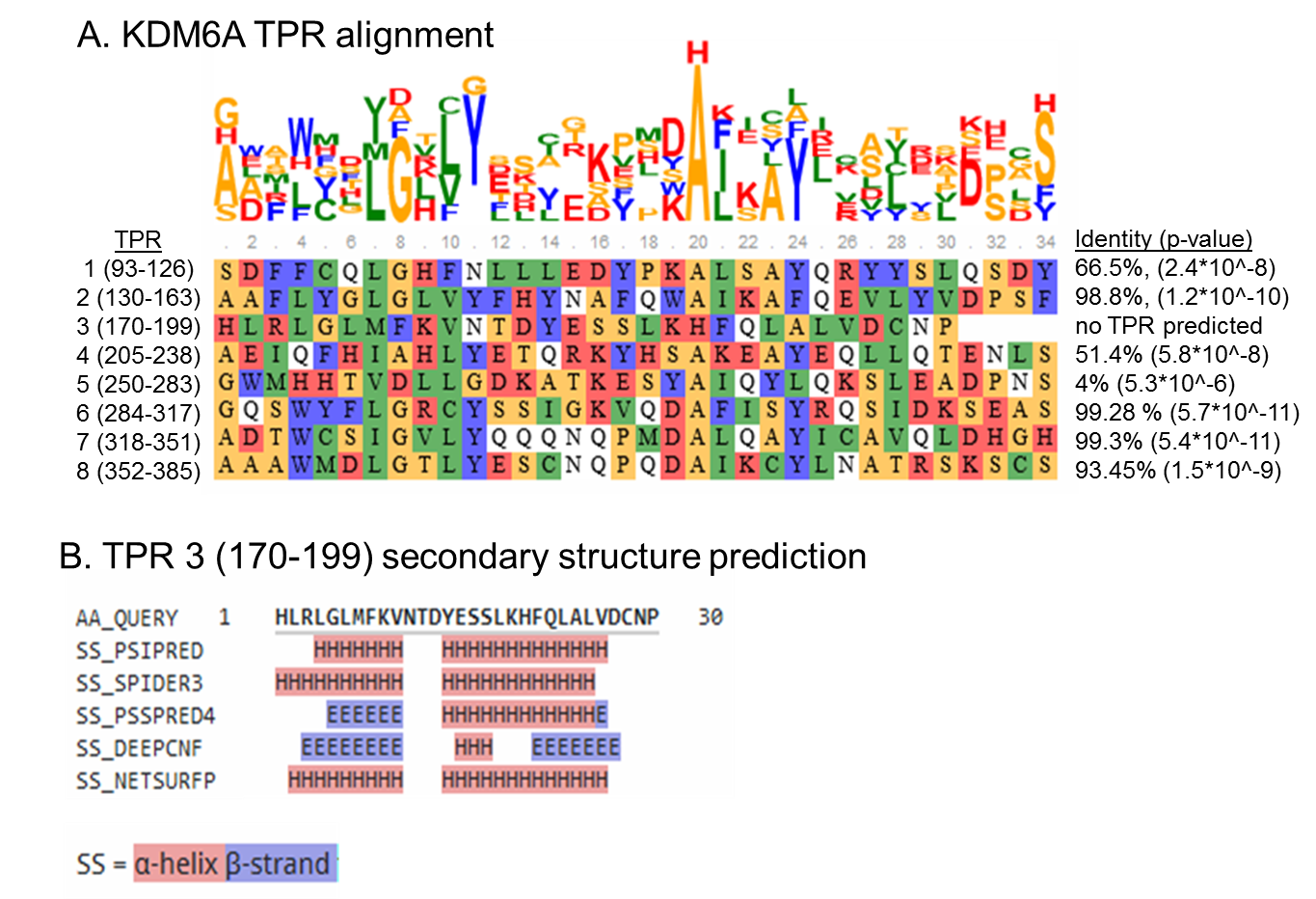


**Bioinformatic analysis of KDM6A tetratricopeptide repeats**. **A.** Alignment of annotated KDM6A TPRs using the *TPRprediction and alignment tool* published in [1]. All annotated KDM6A TPRs with the given amino acid range (see label) were compared for their TPR conservation to the consensus sequence as published in [4] with the most conserved amino acids W4, L7, G8, Y11, A20, Y24, A27 and P30. Identity and p-values are shown on the right hand. The 34 amino acid TPR motif could not be identified for TPR 3 (aa 170-199). Color coding according to CLUSTAL W alignment. **B**. secondary structure prediction of TPR3 using the Quick2D on the MPI Bioinformatics toolkit server [2, 3] indicated a high probability for aa 14-26 to form an α-helix, but aa 1-10 tend to form either an α-helix or a β-strand. Of note, the canonical 34 aa TPR repeating unit forms the crucial helix-turn-helix motif [4]. In general, the N-terminal TPRs 1-4 show less strict conservation towards the canonical TPR sequence compared to the C-terminal TPRs 6-8.

1. Karpenahalli, M.R., A.N. Lupas, and J. Soeding, *TPRpred: a tool for prediction of TPR-, PPR- and SELI-like repeats from protein sequences.* Bmc Bioinformatics, 2007. **8**.

2. McGuffin, L.J., K. Bryson, and D.T. Jones, *The PSIPRED protein structure prediction server.* Bioinformatics, 2000. **16**(4): p. 404-405.

3. Zimmermann, L., et al., *A Completely Reimplemented MPI Bioinformatics Toolkit with a New HHpred Server at its Core.* Journal of Molecular Biology, 2018. **430**(15): p. 2237-2243.

4. Main, E.R.G., et al., *Design of stable alpha-helical arrays from an idealized TPR motif.* Structure, 2003. **11**(5): p. 497-508.
