# Supplementary material for: KDM6A mutations promote acute cytoplasmic DNA release, DNA damage response and mitosis defects
Source: BMC Mol Cell Biol. 2021 Oct 26;22:54. doi: 10.1186/s12860-021-00394-2 (PMC8549169; doi:10.1186/s12860-021-00394-2)
Supplement: Supplementary file 5 — Additional file 5: Figure S2. Activity assay and data analysis. In this section, we describe our data analysis workflow. A. delta (grey shaded area) in fluorescence emission spectrum of eGFP-KDM6A WT lysate before incubation and after incubation with GFP-trap dynabeads. For data analysis, the amount of pulled fluorescent protein was determined by subtracting the eGFP fluorescence emission (470 nm excitation) after incubation with GFP-trap dynabeads from eGFP fluorescence emission before incubation with eGFP-trap dynabeads. Fluorescence intensities in the range of 500–530 nm were integrated subsequently (later referred to as integrated fluorescence). B. Maximum-normalized spectra obtained from crude cell lysate of eGFP, eGFP-KDM6A WT or eGFP-KDM6A Q1133A samples. All fluorescence emission spectra of the substitution variants had a similar profile between 500 and 530 nm. This was used to compare the amount of protein for each variant. Q1133A variant is shown exemplarily for all other substitution variants. C. Fluorescence emission spectra of WT and ΔJmjC. The high signal of ΔJmjC towards the blue end of the spectrum is due to scatter, which could indicate protein instability under the given (low salt) buffer conditions. D. 1–238 ng demethylated product (H3K27me2 peptide, MW = 2945.5 g/mol) was obtained from at least nine data points from three independent experiments, each in triplicates and fitted with a four parameters logistic regression (4-PL) according to Eq. 1: $\mathrm{y}=\mathrm{d}+\frac{\mathrm{a}-\mathrm{d}}{1+{\left(\frac{\mathrm{x}}{\mathrm{c}}\right)}^{\mathrm{b}}}$y=d+a−d1+xcb (1) with y = assay readout absorption, x = amount of product in A., amount of KDM6A species, either by fluorescence in B. or amount of pro [file 12860_2021_394_MOESM5_ESM.docx]

**Figure S2**

A.

B.

C.

D.

E.

F.

G.

H.

I.

**Activity assay and data analysis. A**. Delta (grey shaded area) in fluorescence emission spectrum of eGFP-KDM6A WT lysate before incubation and after incubation with GFP-trap dynabeads. For data analysis, the amount of pulled fluorescent protein was determined by subtracting the eGFP fluorescence emission (470 nm excitation) after incubation with GFP-trap dynabeads from eGFP fluorescence emission before incubation with eGFP-trap dynabeads. Fluorescence intensities in the range of 500-530 nm were integrated subsequently (later referred to as integrated fluorescence). **B**. Maximum-normalized spectra obtained from crude cell lysate of eGFP, eGFP-KDM6A WT or eGFP-KDM6A Q1133A samples. All fluorescence emission spectra of the substitution variants had a similar profile between 500-530 nm. This was used to compare the amount of protein for each variant. Q1133A variant is shown exemplarily for all other substitution variants. **C**. Fluorescence emission spectra of the WT and ΔJmjC variants. The high signal of ΔJmjC variant towards the blue end of the spectrum is due to the scatter, which could indicate protein instability under the given (low salt) buffer conditions. **D**. 1-238 ng demethylated product (H3K27me2 peptide, MW = 2945.5 g/mol) was obtained from at least nine data points from three independent experiments, each in triplicates and fitted with a four parameters logistic regression (4-PL) according to *Eq. 1:*

$\text{y = d + }\frac{\text{a - d}}{{\text{1 + }\left( \frac{\text{x}}{\text{c}} \right)}^{\text{b}}}$ *Eq. 1*

with *y* = absorption, *x* = amount of product in **A**., amount of KDM6A species, either by fluorescence in **B**. or amount of protein in **D**., *a* = background signal, *d* = maximum signal, *b* = slope, *c* = c50, x-value at half maximum y.

The following fit results were obtained: *a = 0.29, b = 1.27, d = 1.73 and c = 62.4 ng*. **E**. The amount of protein was plotted against absorption (shown for KDM6A WT) and fitted accordingly with 4-PL, with *c* being the only open parameter. c50 for WT was determined with 518,000 +/- 56,000 AU. To correct for background and maximum signal fluctuation between experiments, they were normalized to the values obtained from the product curve fit (*a = 0.29, d = 1.73*). **F**. A defined amount of recombinant eGFP (27 kDa) was used to convert the x-axis from fluorescent signal to the amount of protein. The linear dependency was used to calculate the amount of eGFP-KDM6A (181 kDa) per fluorescent unit. The molar integrated fluorescence (500-530 nm) from emission spectra for recombinant eGFP (MW = 26.9 kDa) was determined at 2.34E+18 AU/mol from the relation 1 ng eGFP = 86,900 [AU]. Therefore, 1 ng eGFP-KDM6A WT would yield an integrated fluorescence signal of 1.29E+03 AU (181 kDa MW). **G**. Data from **E**. and **F**. were used to transform the x-Axis with the fluorescent signal into the amount of KDM6A protein. Approximated from **D**., the respective *c50* always corresponds to 62.4 product. This correlation was used to calculate the specific activity for each variant. Given the fixed time of 240 min per assay, the specific activity under these conditions was calculated. The *c50* for all available substitution variants was obtained in the same manner, fixing all parameters except *c*, using it as a relative measure of activity. At least four independent triplicate measurements were obtained for each substitution variant. **H**. absorption for truncated variants, three repeats with triplicates each. Since the truncated variants showed an unusual spectrum with a high scatter fraction at lower wavelengths (see **C**.), a quantification was impossible. Therefore, we qualitatively confirmed, that all truncated variants with a JmjC domain are catalytically active (green boxes) and those without JmjC domain are catalytically dead (red boxes). The distribution of the signal over the background is an evidence for activity in all truncated variants with a JmjC domain. ΔJmjC and TPR do not exceed assay background levels, while WT, ΔIDR, ΔTPR and JmjC variants are all above background. **I**. Activity of the T726K variant is dependent on post expression time. WT activity does not change significantly between 24 h (black) to 48 h (grey), whereas in T726K activity is slightly lower than WT after 24 h (blue) and strongly reduced after 48 h (cyan) when comparing c50-values
