# Supplementary material for: KDM6A mutations promote acute cytoplasmic DNA release, DNA damage response and mitosis defects
Source: BMC Mol Cell Biol. 2021 Oct 26;22:54. doi: 10.1186/s12860-021-00394-2 (PMC8549169; doi:10.1186/s12860-021-00394-2)
Supplement: Supplementary file 6 — Additional file 6: Figure S3. Control experiments for antibody staining specificity and endogenous KDM6A protein levels. SW-1710 and T-24 cells were transfected with KDM6A WT or ΔIDR variants and stained with two different antibodies (sc-514,859, Santa Cruz biotechnology and CST-33510, Cell Signaling) raised against epitopes within the KDM6A central region (IDR) and 2nd antibody labeled with AbStar Red (Abberior). Clearly, both antibodies (in red channel) detect transfected KDM6A WT protein (very strong signals in green channel), but not the ΔIDR variant. The untransfected control cells show endogenous KDM6A in both cell lines stained with both KDM6A antibodies. [file 12860_2021_394_MOESM6_ESM.docx]

**Figure S3**

**
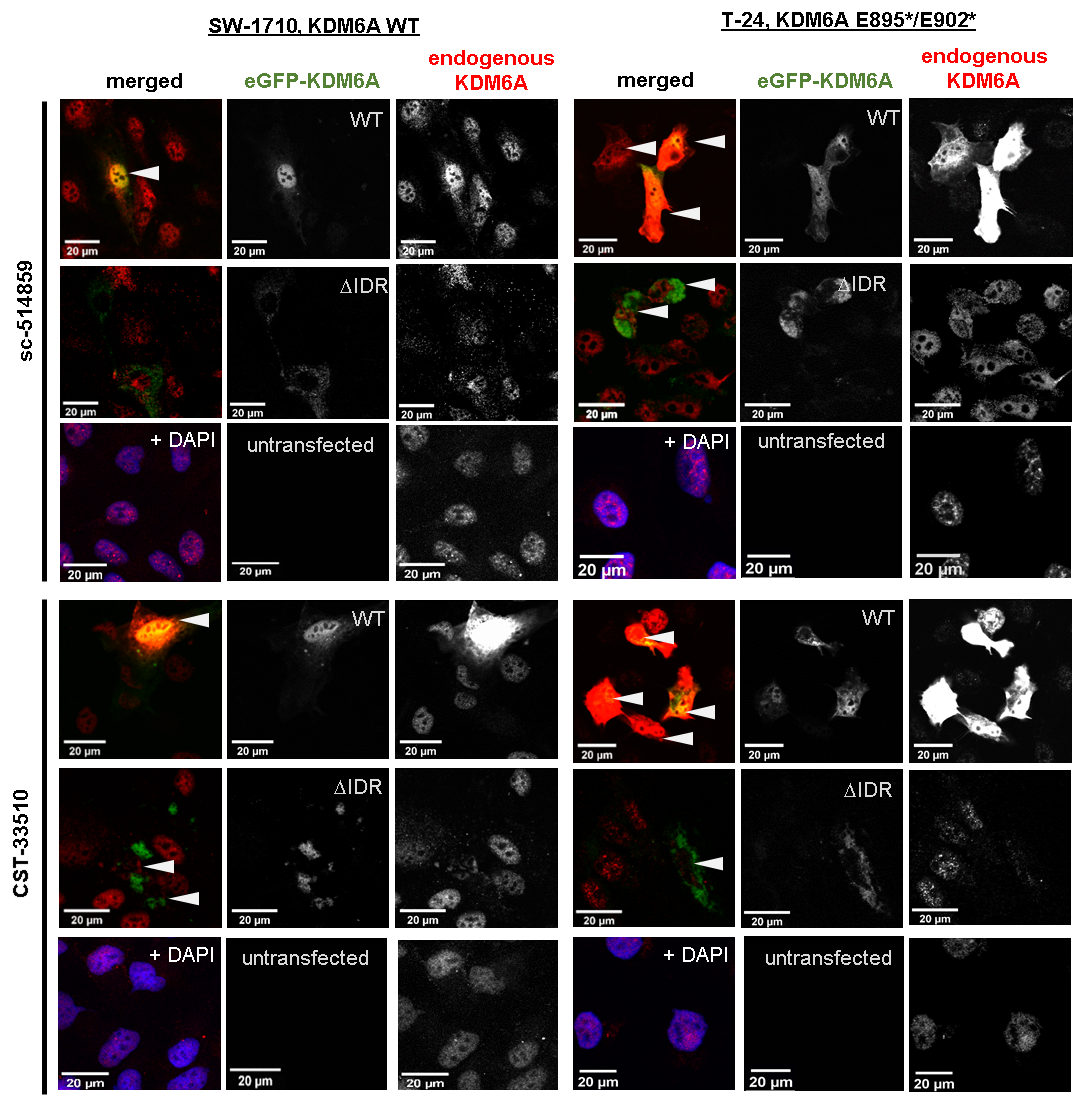
**

**Control experiments for antibody staining specificity and endogenous KDM6A protein levels.** SW-1710 and T-24 cells were transfected with KDM6A WT or ΔIDR variants and stained with two different antibodies (sc-514859, Santa Cruz biotechnology and CST-33510, Cell Signaling) raised against epitopes within the KDM6A central region (IDR) and 2^nd^ antibody labeled with AbStar Red (Abberior). Clearly, both antibodies (in red channel) detect transfected KDM6A WT protein (very strong signals in green channel), but not the ΔIDR variant. The untransfected control cells show endogenous KDM6A in both cell lines with both KDM6A antibodies.
