# Supplementary material for: KDM6A mutations promote acute cytoplasmic DNA release, DNA damage response and mitosis defects
Source: BMC Mol Cell Biol. 2021 Oct 26;22:54. doi: 10.1186/s12860-021-00394-2 (PMC8549169; doi:10.1186/s12860-021-00394-2)
Supplement: Supplementary file 7 — Additional file 7: Figure S4. Localization study of KDM6A variants in normal and invasive urothelial (cancer) cells. A. KDM6A substitution and deletion variants are localized in the cytoplasm and nucleus in T-24 cells. Substitutions cause mild effects, whereas deletion variants cause stronger cellular responses such as DNA release and mitotic errors. B. Representative KDM6A deletion variants are localized in the cytoplasm and nucleus in HBLAK. Two different phenotypes have been observed: a milder phenotype (majority of cells) and a phenotype (minority of cells) which reproducibly causes stronger cellular responses such as DNA release and mitotic errors. DAPI counterstaining of the nucleus and the respective overlay of both channels (DAPI in blue and GFP in green) is displayed. Images are taken in 2048 × 2048 resolution using a widefield microscope Olympus FX81 with 60x oil objective with post-processing using HuygensPro20.10. Effects of KDM6A variants in SW-1710 cells is shown in Fig. 2. [file 12860_2021_394_MOESM7_ESM.docx]

**Figure S4**

**
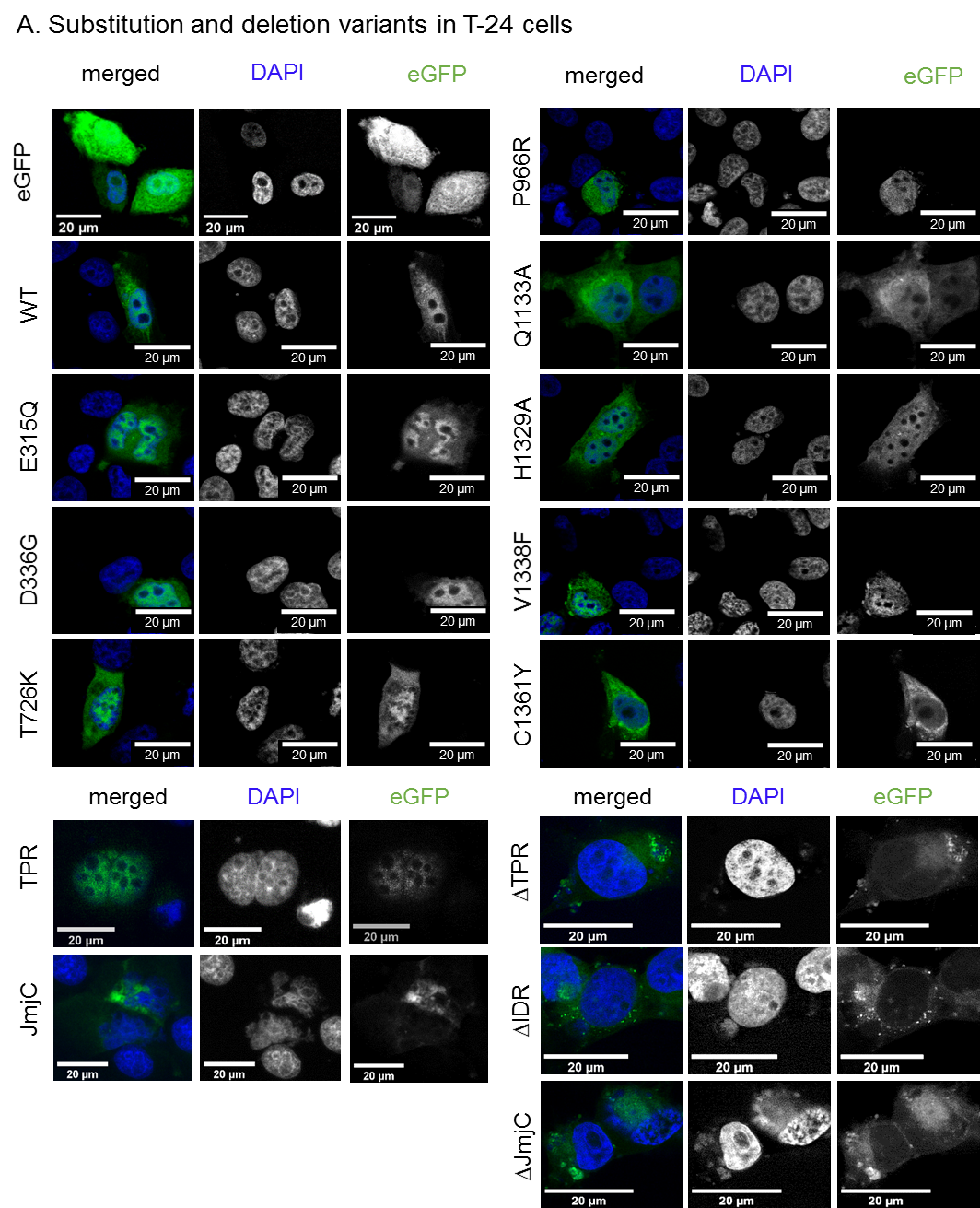
**

**
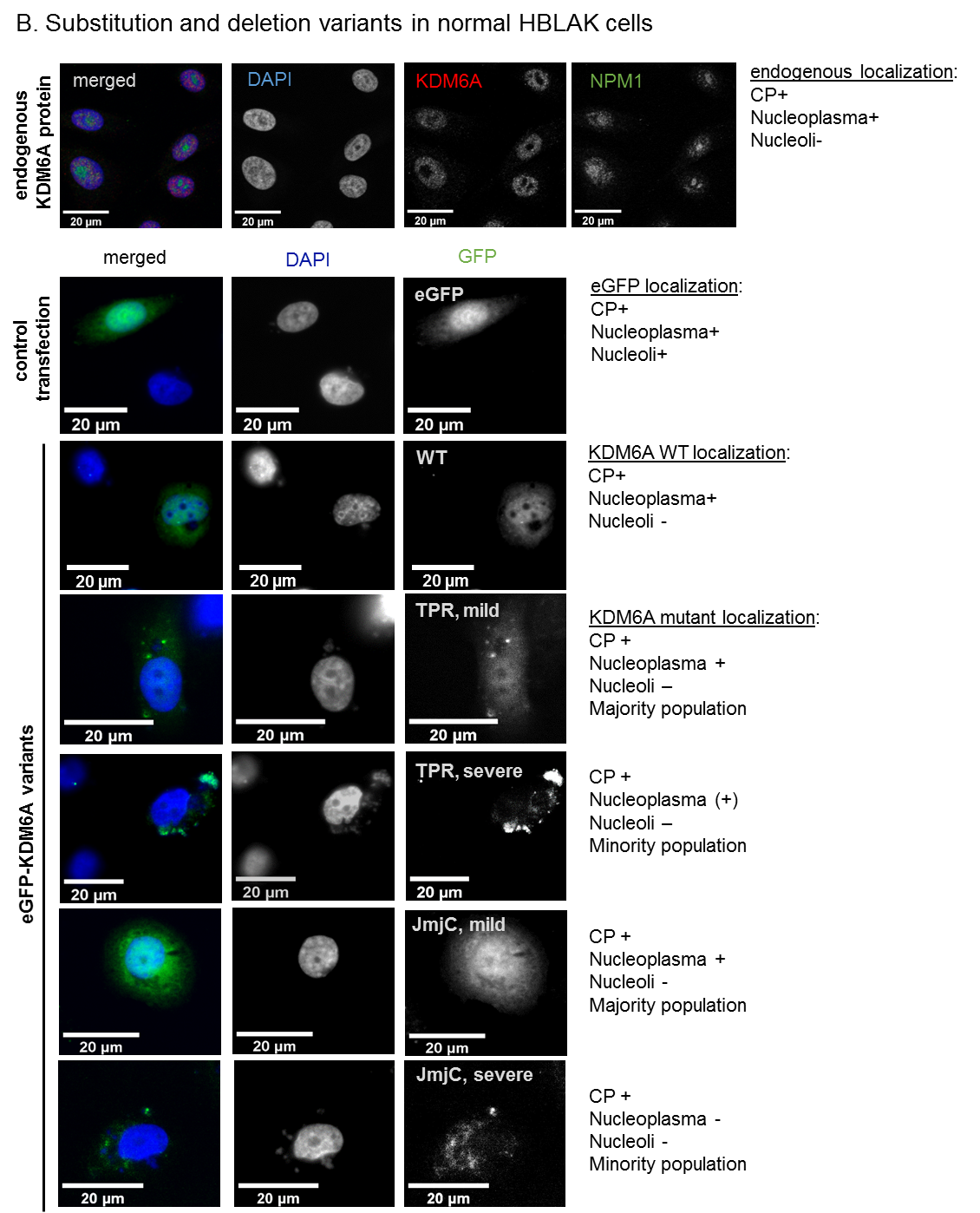
**

**Localization study of KDM6A variants in normal and invasive urothelial (cancer) cells. A.** KDM6A substitution and deletion variants are localized in the cytoplasm and nucleus in T-24 cells. Substitutions cause mild effects, whereas deletion variants cause stronger cellular responses such as DNA release and mitotic errors. **B.** representative KDM6A deletion variants are localized in the cytoplasm and nucleus in HBLAK. Two different phenotypes have been observed: a milder phenotype (majority of cells) and a phenotype (minority of cells) which reproducibly cause stronger cellular responses such as DNA release and mitotic errors.

DAPI counterstaining of the nucleus and the respective overlay of both channels (DAPI in blue and GFP in green) is displayed. Images are taken in 2048x2048 resolution using the widefield microscope Olympus FX81 with 60x oil objective with post-processing using HuygensPro20.10. Effects of KDM6A variants in SW-1710 cells is show in **Figure 2**.
