# Supplementary material for: KDM6A mutations promote acute cytoplasmic DNA release, DNA damage response and mitosis defects
Source: BMC Mol Cell Biol. 2021 Oct 26;22:54. doi: 10.1186/s12860-021-00394-2 (PMC8549169; doi:10.1186/s12860-021-00394-2)
Supplement: Supplementary file 8 — Additional file 8: Figure S5. Western Blot control experiments and raw blots. A. eGFP-KDM6A TPR whole cell lysate and the corresponding IP fraction was separated on a Western Blot and stained with eGFP (CST) or RBBP5 (CST) antibodies. RBBP5 precipitated with eGFP-KDM6A TPR. B. Dynabeads washing control on same membrane, washed with 3x RIPA like buffer (RLB). While KDM6A WT pulls a clearly visible RBBP5 band and a low WDR5 band, both are undetectable in eGFP-transfected cells alone (negative control). C. Raw blots from Fig. 3. Lysate and Co-IP for selected KDM6A variants and eGFP. IP and corresponding lysate lanes were also on the same blot. All experimental conditions for the blots presented were kept constant. D. Raw blots from Fig. 1B/D. Protein lanes shown in main figures are in red boxes. The PageRuler Plus Prestained protein ladder (Thermo Fisher) was used for orientation. [file 12860_2021_394_MOESM8_ESM.docx]

**Figure S5**

**
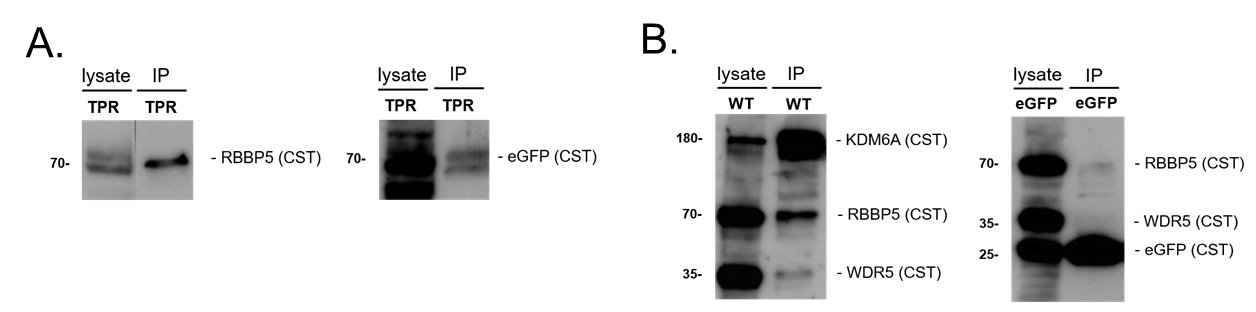
**

**
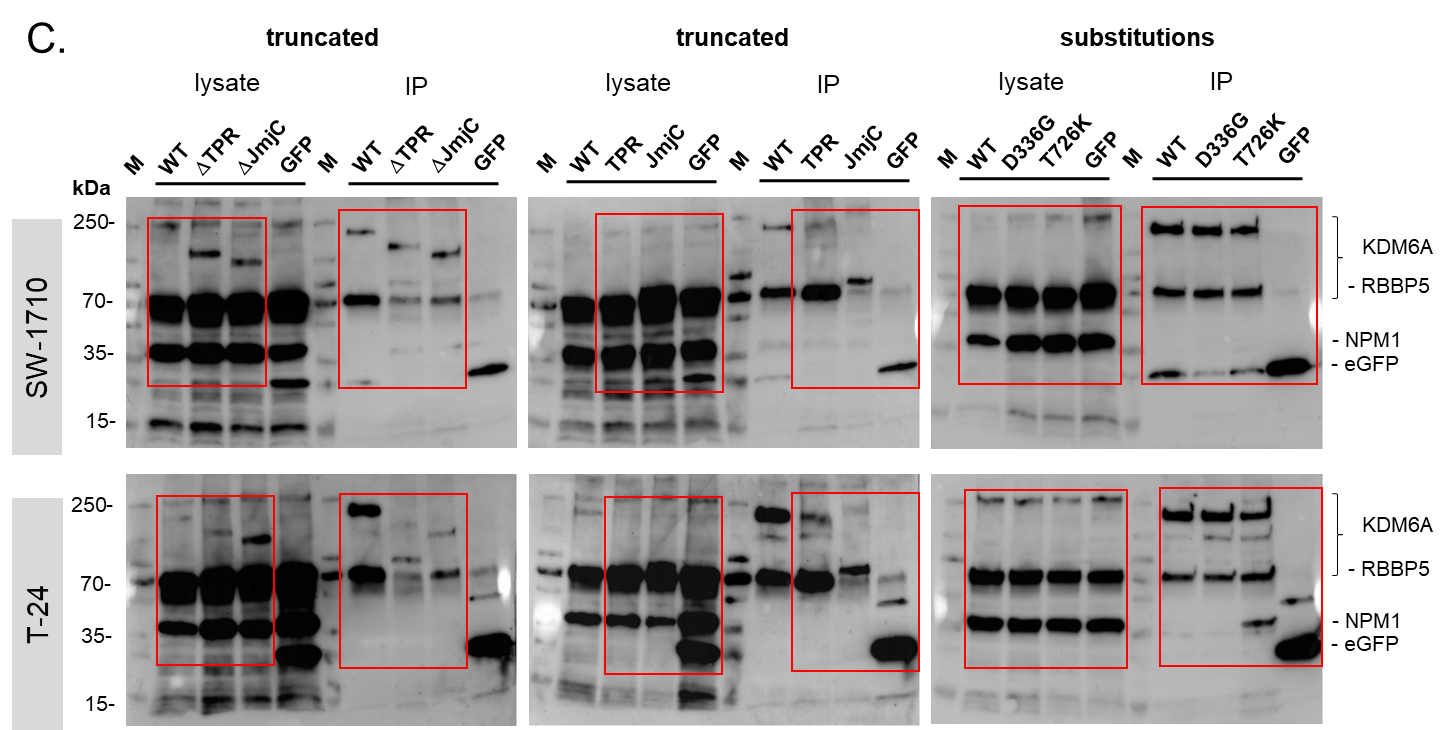
**

**
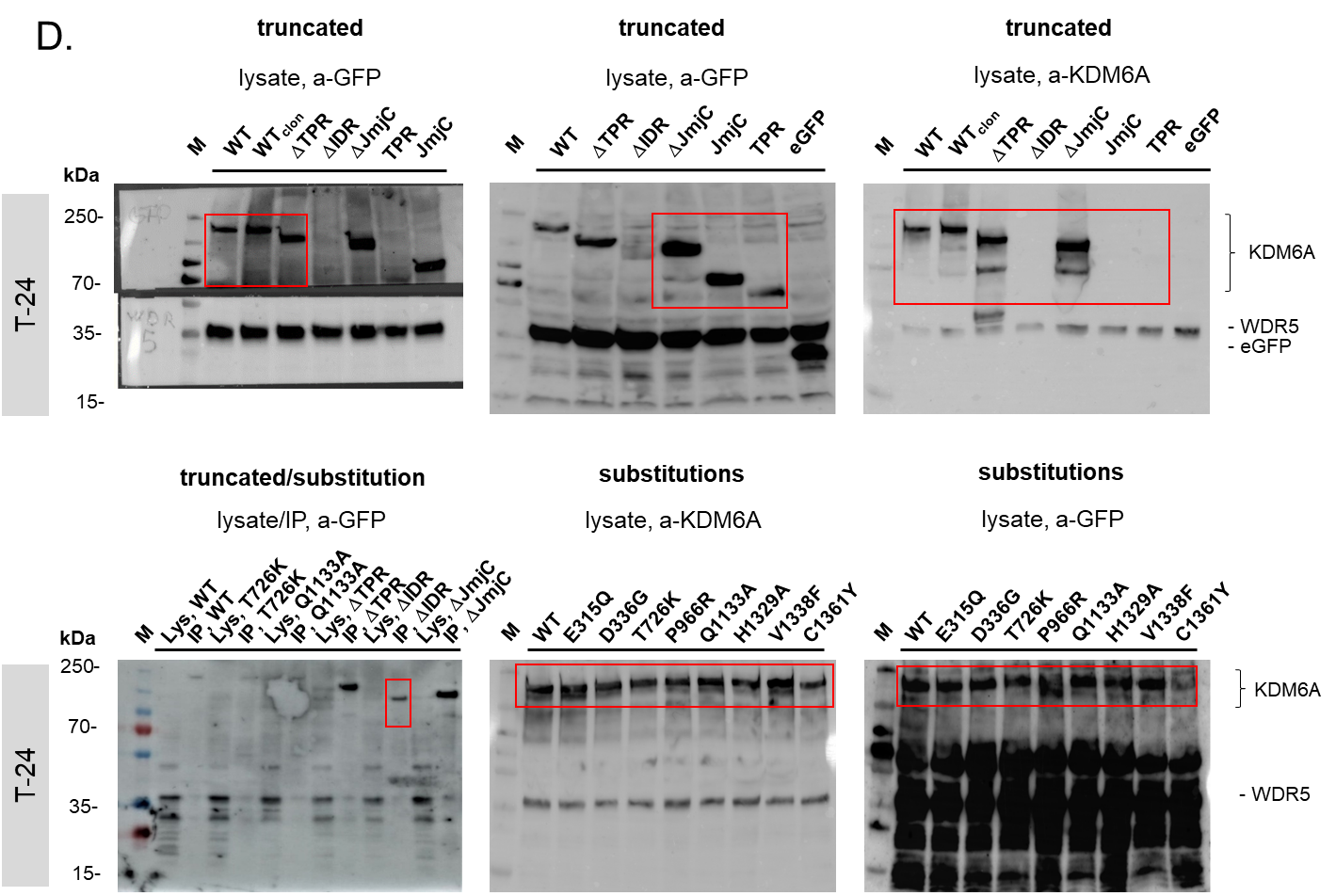
**

**Western Blot control experiments and raw blots. A**. eGFP-KDM6A TPR whole cell lysate and the corresponding IP fraction was separated on a Western Blot and stained with eGFP (CST) or RBBP5 (CST) antibodies. RBBP5 precipitated with eGFP-KDM6A TPR. **B**. Dynabeads washing control on same membrane, washed with 3x RIPA like buffer (RLB). While KDM6A WT pulls a clearly visible RBBP5 band and a low WDR5 band, both are undetectable in eGFP-transfected cells alone (negative control). **C.** Raw blots from Figure 3. Lysate and Co-IP for selected KDM6A variants and eGFP. IP and corresponding lysate lanes were also on the same blot. All experimental conditions for the blots presented were kept constant. **D**. Raw blots from Figure 1B/D. Protein lanes shown in main figures are in red boxes. The PageRuler Plus Prestained protein ladder (Thermo Fisher) was used for orientation.
