# Supplementary material for: KDM6A mutations promote acute cytoplasmic DNA release, DNA damage response and mitosis defects
Source: BMC Mol Cell Biol. 2021 Oct 26;22:54. doi: 10.1186/s12860-021-00394-2 (PMC8549169; doi:10.1186/s12860-021-00394-2)
Supplement: Supplementary file 9 — Additional file 9: Figure S6. Pathway enrichment analysis of MS data from KDM6A-tagGFP2 cell lines. A. Data from quadruplicates of three independent experiments of two different KDM6A-tagGFP2 cell lines using the reactome database. MS data sets were applied to the reactome analysis database. The resulting output shows significantly enriched pathways. The five most interesting pathways are depicted: cell cycle, chromatin organization, DNA repair, RNA metabolism including rRNA processing and protein metabolism including ribosome biogenesis and post-translational modification of histones. B. Top ten proteins identified by MS in KDM6A-tagGFP2 stable cell lines VM-CUB1 and RT-112. Among the top ten proteins in KDM6A pull down and subsequent MS, Nucleophosmin (NPM1), Nucleolin, ribosomal subunits 40S and 60S, as well as histone variants, are detectable. Mutational KDM6A and KMT2C/D status and experiments with RT-112 and VM-CUB1 have been recently published [18]. [file 12860_2021_394_MOESM9_ESM.docx]

**Figure S6**

**A.**

**
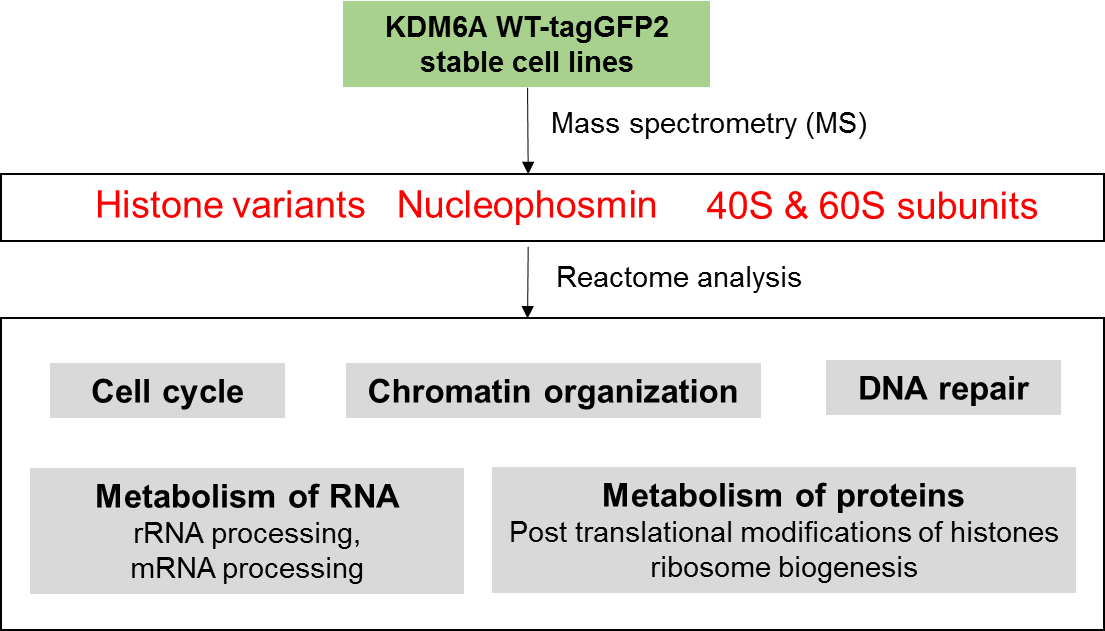
**

**B.**

| **#** | **p value** | **Protein names** |
| --- | --- | --- |
| **1** | **7.71E-08** | **KDM6A** |
| **2** | **6.19E-07** | **NPM1** |
| 3 | 4.31E-06 | 60S ribosomal protein L10a |
| 4 | 5.53E-05 | Histone H2A types A/B/C |
| 5 | 2.66E-05 | 60S ribosomal protein L18a |
| 6 | 7.40E-05 | Histones H4 |
| 7 | 4.88E-04 | 60S ribosomal protein L14 |
| 8 | 4.23E-04 | Histones H2A.V H2A.Z |
| 9 | 3.80E-04 | Histone H3.3 |
| 10 | 2.63E-04 | Nucleolin |

**Pathway enrichment analysis of MS data from KDM6A-tagGFP2 cell lines. A.** Data from quadruplicates of three independent experiments of two different KDM6A-tagGFP2 cell lines using the reactome database. MS data sets were applied to the reactome analysis database. The resulting output shows significantly enriched pathways. The five most interesting pathways are depicted: cell cycle, chromatin organization, DNA repair, RNA metabolism including rRNA processing and protein metabolism including ribosome biogenesis and post-translational modification of histones. **B**. Top ten proteins identified by MS in KDM6A-tagGFP2 stable cell lines VM-CUB1 and RT-112. Among the top ten proteins in KDM6A pull down and subsequent MS, Nucleophosmin (NPM1), Nucleolin, ribosomal subunits 40S and 60S, as well as histone variants, are detectable. Mutational KDM6A and KMT2C/D status and experiments with RT-112 and VM-CUB1 have been recently published [1]

References

1. Lang, A., et al., *Contingencies of UTX/KDM6A Action in Urothelial Carcinoma.* Cancers, 2019. **11**(4).
