# Supplementary material for: KDM6A mutations promote acute cytoplasmic DNA release, DNA damage response and mitosis defects
Source: BMC Mol Cell Biol. 2021 Oct 26;22:54. doi: 10.1186/s12860-021-00394-2 (PMC8549169; doi:10.1186/s12860-021-00394-2)
Supplement: Supplementary file 10 — Additional file 10: Figure S7. Cellular back mapping identifies co-localizing protein populations at extranuclear DNA segments. A. images shown in Fig. 4, all channels. B. Analysis of KDM6A TPR and NPM1: Cellular back mapping identifies a colocalizing population (orange) and free KDM6A TPR (green) and NPM1 (red) at cytoplasmic DNA segments. C. scatterplot analysis and cellular back mapping of KDM6A variants and DAPI as indicator of KDM6A-DNA complexes. In KDM6A WT scatterplot we identified four populations: 1 = cytoplasmic KDM6A (without DAPI) and 4 = DAPI (without KDM6A), as well as, populations #2 and #3, which resemble different ratios of KDM6A-DNA complexes. Population 2 is located at the lamina and in nucleolar puncta, and population 3 is located within the nucleoplasm. Substitution variant T726K shows minor differences in populations #2 and #4, as the lamina in #2 is less clearly visible, but strongly enriched in population #4. Contour scatterplot of JmjC or TPR with DAPI identified only three populations, with population #1 being absent. Back mapping clearly indicates compartment-wise localization of different DAPI-KDM6A JmjC or TPR compositions. [file 12860_2021_394_MOESM10_ESM.docx]

**Figure S7**

**
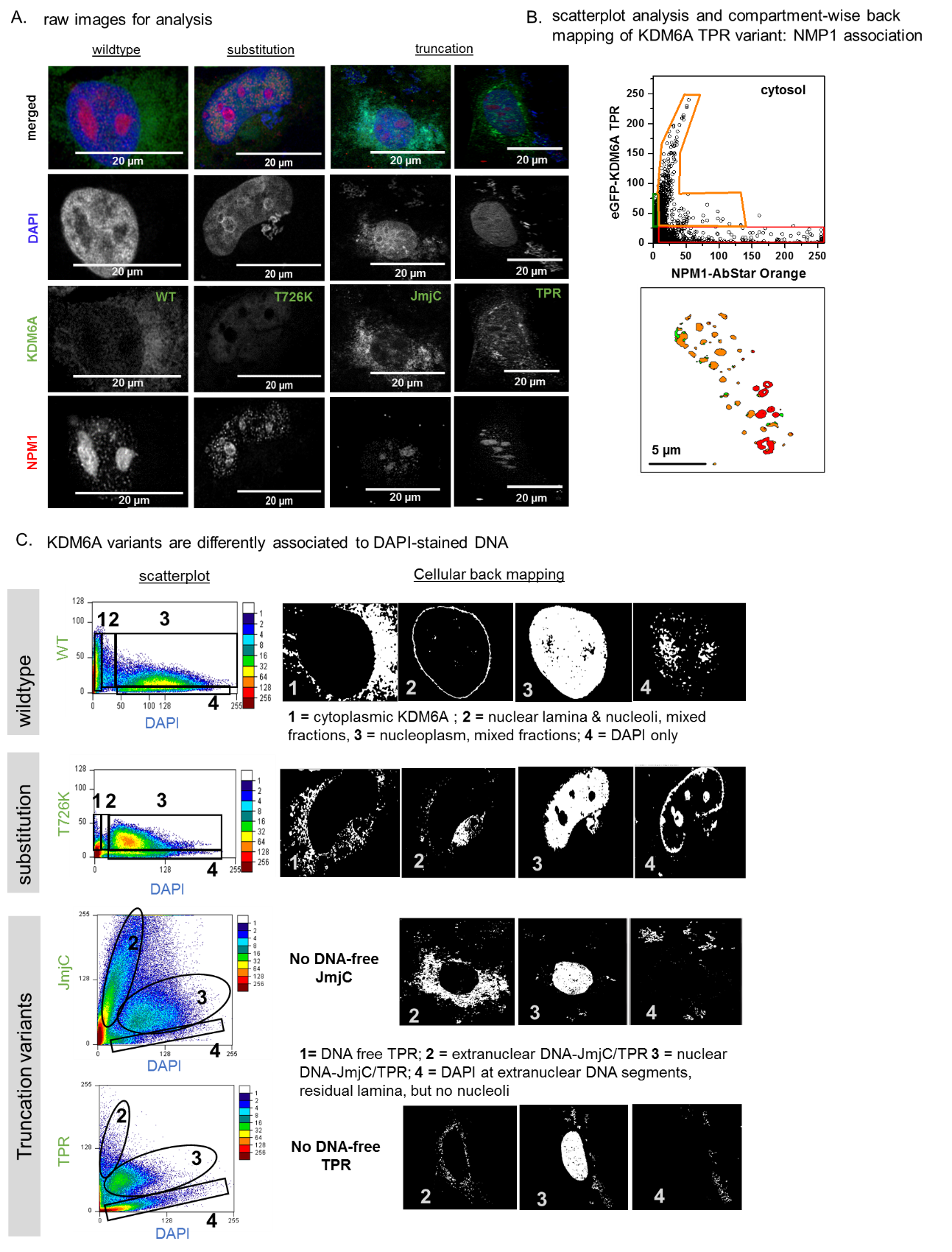
**

**Cellular back mapping identifies co-localizing protein populations at extranuclear DNA segments**. **A.** images shown in **Figure 4**, all channels**. B**. Analysis of KDM6A TPR and NPM1: Cellular back mapping identifies a colocalizing population (orange) and free KDM6A TPR (green) and NPM1 (red) at cytoplasmic DNA segments. **C.** scatterplot analysis and cellular back mapping of KDM6A variants and DAPI as indicator of KDM6A-DNA complexes. In KDM6A WT scatterplot we identified four populations: 1 = cytoplasmic KDM6A (without DAPI) and 4 = DAPI (without KDM6A), as well as, populations #2 and #3, which resemble different ratios of KDM6A-DNA complexes. Population 2 is located at the lamina and in nucleolar puncta, and population 3 is located within the nucleoplasm. Substitution variant T726K shows minor differences in populations#2 and #4, as the lamina in #2 is less clearly visible, but strongly enriched in population #4. Contour scatterplot of JmjC or TPR with DAPI identified only three populations, with population #1 being absent. Back mapping clearly indicates compartment-wise localization of different DAPI-KDM6A JmjC or TPR compositions.
