# Supplementary material for: KDM6A mutations promote acute cytoplasmic DNA release, DNA damage response and mitosis defects
Source: BMC Mol Cell Biol. 2021 Oct 26;22:54. doi: 10.1186/s12860-021-00394-2 (PMC8549169; doi:10.1186/s12860-021-00394-2)
Supplement: Supplementary file 11 — Additional file 11: Figure S8. Distribution and expression levels of p-γH2A.X and RAD51 after KDM6A overexpression and endogenous localization of KDM6A during mitosis. A. p-γH2A.x protein level in untransfected and KDM6A variants transfected T-24 cells. Note, that even in strongly overexpressed KDM6A WT cells, p-γH2A.X was not increased, but strongly so in KDM6A JmjC and ΔTPR cells. B. Endogenous localization of KDM6A (CST antibody, 2nd antibody goat-anti-rabbit AbStar red) as imaged with Leica STED SP8 in HBLAK cells. For STED, the sample was depleted with 35% of 775 nm laser. C. RAD51 proteins were detected at a persisting chromatin bridge of two daughter cells, with cell 1 being KDM6A ΔTPR positive. Image was taken with Abberior STED, excitation laser 640 nm 5% and depletion laser 775 nm with 40%. [file 12860_2021_394_MOESM11_ESM.docx]

**Figure S8**

**
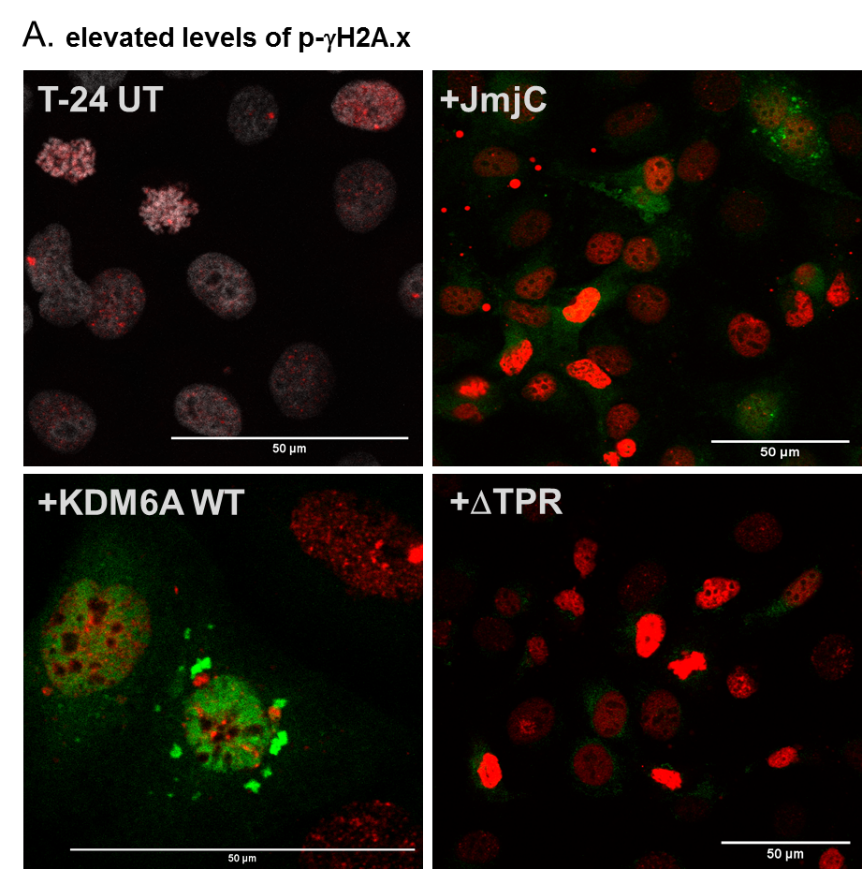
**


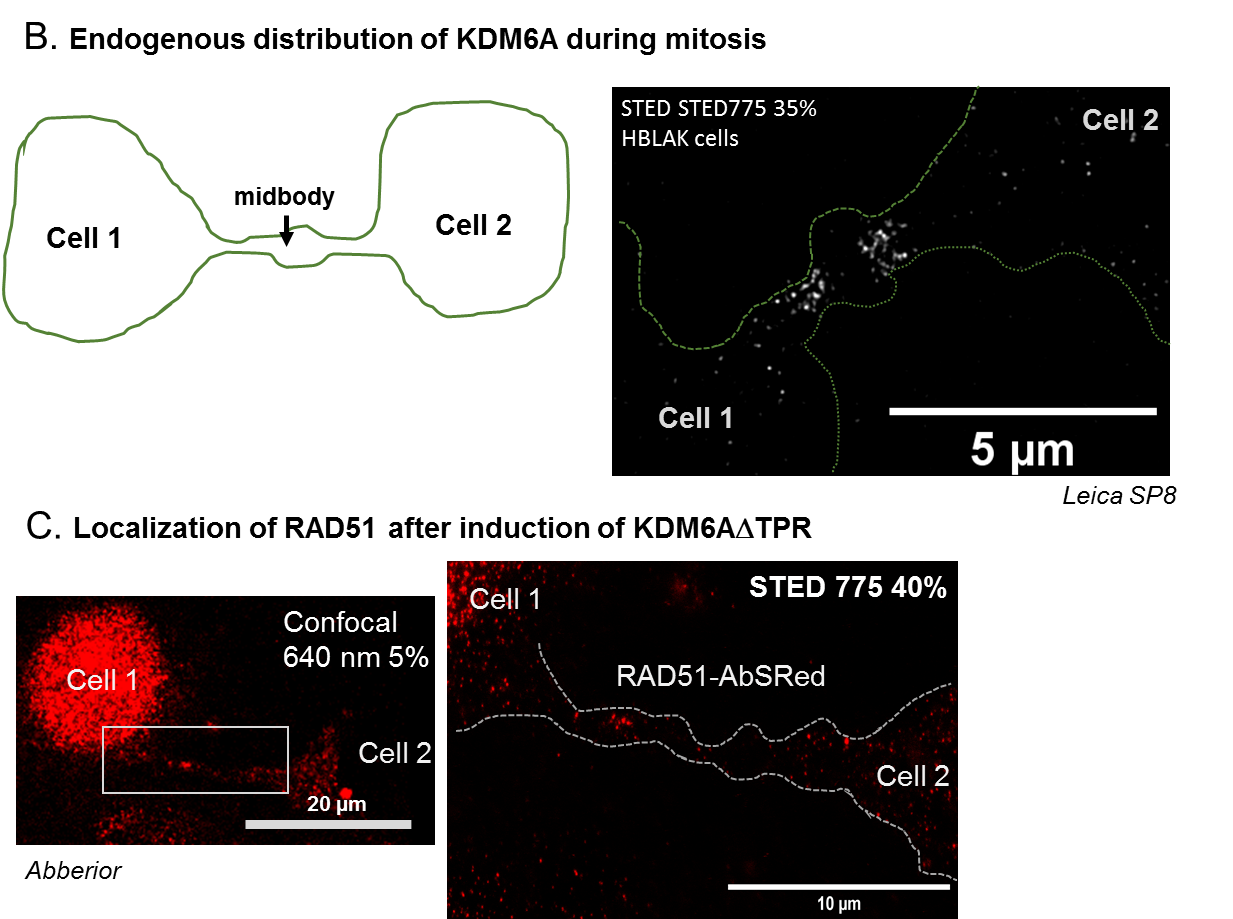


**Distribution and expression levels of p-γH2A.x and RAD51 after KDM6A overexpression and endogenous localization of KDM6A during mitosis**. **A**. p-γH2A.x protein level in untransfected and KDM6A variants transfected T-24 cells. Note, that even in strongly overexpressed KDM6A WT cells, p-γH2A.x was not increased, but strongly in KDM6A JmjC and ΔTPR cells. **B**. endogenous localization of KDM6A (CST antibody, 2^nd^ antibody goat-anti-rabbit AbStar red) as imaged with Leica STED SP8 in HBLAK cells. For STED, the sample was depleted with 35% of 775 nm laser. **C**. RAD51 proteins were detected at a persisting chromatin bridge of two daughter cells, with cell 1 being KDM6A ΔTPR positive. The image was taken with Abberior STED, excitation laser 640 nm 5% and depletion laser 775 nm with 40%.
